# Optimization strategies for crystal orientation in antimony-based chalcogenide thin-film solar cells

**DOI:** 10.1186/s40580-026-00557-x

**Published:** 2026-06-04

**Authors:** Xuefeng Chen, Xueling Chen, Hangrui Zhang, Yingying Mo, Xiaomin Wang, Xudong Xiao, Jianmin Li

**Affiliations:** 1 Key Laboratory of Artificial Micro- and Nano-Structures of Ministry of Education, School of Physics and Technology, Wuhan University, Wuhan, People’s Republic of China; 2 Hubei Key Laboratory of Plasma Chemistry and Advanced Materials, School of Materials Science and Engineering, Wuhan Institute of Technology, Wuhan, People’s Republic of China

**Keywords:** Antimony chalcogenides, Thin-film solar cells, Crystal orientation regulation, Benign grain boundaries, Growth kinetics

## Abstract

Antimony chalcogenides are highly promising thin-film photovoltaic materials. However, their quasi-one-dimensional structure inherently causes severe transport anisotropy. The thermodynamically stable [hk0] horizontal orientation induces van der Waals barriers that hinder carrier transport, whereas the kinetically favorable [hk1] vertical orientation constructs efficient charge pathways and dangling-bond-free “benign grain boundaries”. Focusing on the thermodynamic and kinetic competition mechanisms during film growth, this review systematically summarizes recent optimization strategies for inducing the [hk1] preferred orientation. Four core approaches are highlighted: solvent and precursor engineering, deposition parameter optimization, interface and substrate engineering, and post-treatment reconstruction. Finally, we delineate the “structure-process-performance” relationship and provide perspectives on deep-level defect passivation, heterojunction band engineering, and flexible, large-area applications, aiming to guide the fabrication of high-efficiency antimony-based solar cells approaching their theoretical limit.

**Graphical abstract:**

**Figure:**
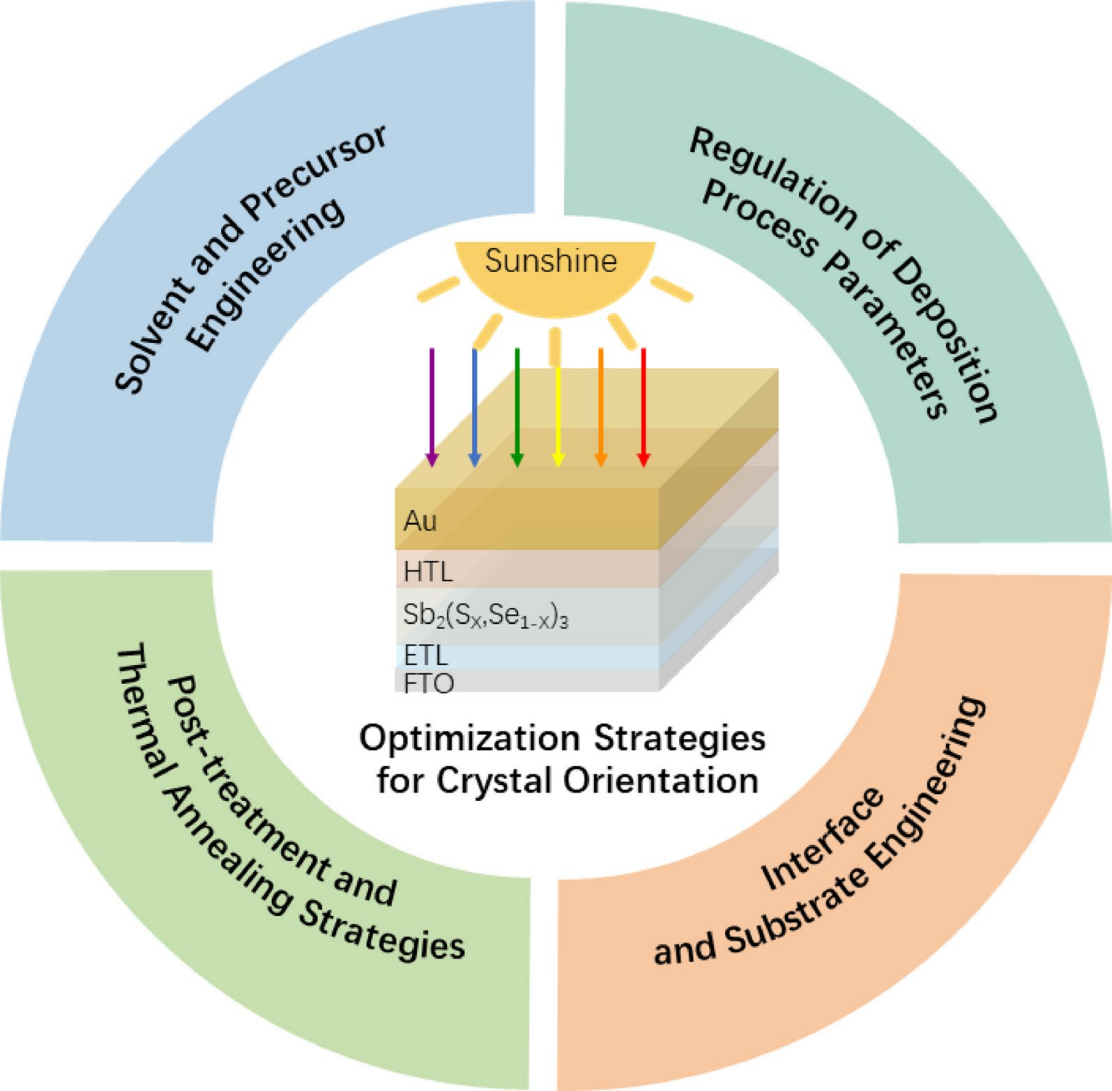
Focusing on the thermodynamic and kinetic competition mechanisms during film growth, this review systematically summarizes four core optimization strategies for inducing the [hk1] preferred orientation, aiming to guide the fabrication of high-efficiency antimony-based solar cells.

## Introduction

**Fig. 1:**
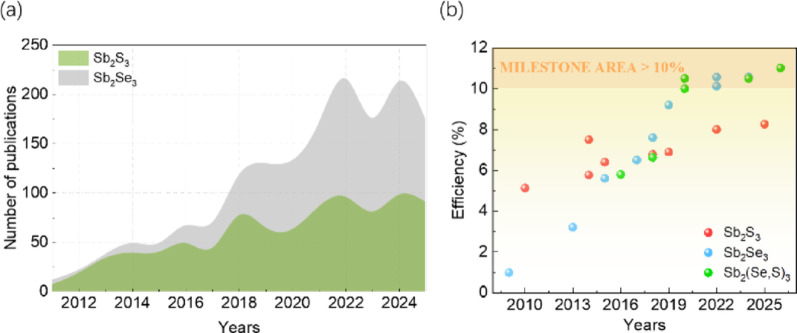
**a** A stacked bar chart showing the annual publication count related to Sb_2_S_3_ and Sb_2_Se_3_ solar cells in the Web of Science database from 2011 to 2025, reflecting a continuous increase in research interest within this field. **b** The evolution of power conversion efficiency (PCE) for antimony-based chalcogenide solar cells (Sb_2_S_3_ [12–19], Sb_2_Se_3_ [11, 20–27], and Sb_2_(S,Se)_3_ [7, 28–31]), with data current as of January 2026. The chart illustrates the technological progress from early low-efficiency stages to the breakthrough of exceeding 10%

With the continuous growth of global energy demand and increasingly severe environmental issues, the development of low-cost, high-efficiency, and environmentally friendly photovoltaic technologies has become a frontier hotspot in scientific research. Among numerous emerging thin-film photovoltaic materials, antimony-based chalcogenides (primarily including Sb_2_S_3_, Sb_2_Se_3_, and their alloy Sb_2_(S,Se)_3_) have attracted widespread attention due to their unique physicochemical properties, with academic research in related fields showing an explosive growth trend in recent years (Fig. 1a) [1, 2]. These materials are abundant in the earth’s crust, have low toxicity, exhibit excellent long-term stability [3, 4], possess suitable direct bandgaps (1.1–1.7 eV), and feature extremely high absorption coefficients (> 10^5^ cm^−1^) [5, 6], making them highly promising candidates for next-generation photovoltaic absorber layers. Driven by in-depth investigations, the power conversion efficiency (PCE) records of antimony-based thin-film solar cells have been continuously refreshed. Notably, the efficiency of Sb_2_(S,Se)_3_ solar cells has successfully surpassed the 10% milestone [7], with the current maximum efficiency reaching 11.02% (Fig. 1b) [8]. However, there is still a significant gap compared to their Shockley-Queisser theoretical limit (> 30%) [9]. The key constraint to further performance enhancement lies in an insufficient understanding of their unique crystal structure and anisotropic carrier transport mechanisms [10, 11].

Distinct from traditional silicon (3D covalent networks) [32] or perovskite (3D ionic lattices) [33, 34] materials, antimony-based chalcogenides belong to the orthorhombic crystal system and possess a special quasi-one-dimensional (Q1D) ribbon-like crystal structure [35–38]. Their lattice consists of [Sb_4_X_6_]_n_ (X = S, Se) nanoribbons extending along a specific axis; atoms within the ribbons are connected by strong covalent bonds, while the ribbons are stacked via weak van der Waals forces [39, 40]. This structural anisotropy leads to significant differences in electrical properties: carrier mobility is high along the covalent bond direction (i.e., [hk1] orientation), whereas transport across the van der Waals gaps (i.e., [hk0] orientation) requires overcoming potential barriers and relies mainly on inefficient hopping conduction mechanisms [41]. Furthermore, crystal orientation directly determines the electronic activity of grain boundaries; boundaries parallel to the [Sb_4_X_6_]_n_ chain growth typically contain no dangling bonds, manifesting as “benign grain boundaries” that effectively suppress non-radiative recombination [42]. Therefore, inducing the formation of [hk1] orientation favorable for longitudinal carrier transport while suppressing the thermodynamically more stable [hk0] orientation has become one of the core scientific issues in the antimony-based photovoltaic field [13, 43].

However, achieving this goal faces complex physicochemical challenges. From a thermodynamic perspective, the (hk0) planes parallel to the nanoribbon axis have the lowest surface energy, causing crystals to naturally tend towards aligning parallel to the substrate [44]. To overcome this intrinsic tendency, kinetic regulation means must be introduced to break the thermodynamic equilibrium during nucleation and growth [43]. In recent years, centering on this “thermodynamic-kinetic competition mechanism”, researchers have conducted extensive exploration into thin-film preparation processes, covering precursor solution chemistry [45], physical vapor deposition parameters [24], interface/substrate engineering [46], and post-treatments [47].

This article aims to systematically review the latest research progress in crystal orientation regulation of antimony-based chalcogenide thin films. First, starting from the crystal structure, we deeply elaborate on the bonding characteristics of the quasi-one-dimensional structure and their anisotropic impact mechanisms on carrier transport and defect behavior. Next, from the perspective of nucleation and growth theory, we analyze the competitive laws between surface energy minimization (thermodynamic dominance) and nucleation growth (kinetic dominance). Furthermore, we focus on summarizing and categorizing existing orientation optimization strategies, primarily including the following four categories:

1. Solvent and Precursor Engineering: Regulating liquid-phase reaction kinetics through ion-mediated processes, compositional optimization, and chemical coordination.
2. Deposition Process Parameter Regulation: Controlling the adsorption and migration behavior of gas-phase molecules through temperature, rate, and vapor composition.
3. Interface and Substrate Engineering: Achieving orientation regulation via lattice-matched epitaxy, seed layer induction, and chemical modification of interlayers.
4. Post-Treatment Strategies: Reconstructing the microscopic structure of thin films through thermal field regulation, flux-assisted recrystallization, and ion penetration.

Through a comprehensive analysis of the aforementioned strategies, this article aims to elucidate the structure-process-property relationships, providing theoretical guidance and technical reference for the future fabrication of antimony-based thin-film photovoltaic devices with low defect density and high performance.

## Crystal structure anisotropy and carrier transport mechanisms

### Quasi-one-dimensional crystal structure and bonding anisotropy

Antimony-based chalcogenides (Sb_2_S_3_, Sb_2_Se_3_, Sb_2_(S,Se)_3_) crystallize in the orthorhombic system (space group *Pnma*), characterized by a distinct quasi-one-dimensional (Q1D) ribbon-like structure [48, 49]. Unlike traditional three-dimensional covalent semiconductors or layered two-dimensional materials, their crystal lattice is composed of [Sb_4_X_6_]_n_ (X = S, Se) nanoribbons extending along a specific axis. These nanoribbons are stacked via weak interactions, resulting in a highly anisotropic bonding system [50].

Along the [001] direction (c-axis), Sb and S/Se atoms are connected by strong covalent bonds, forming robust one-dimensional chains. In this direction, the electron wave functions are highly delocalized, constituting efficient channels for carrier transport. In the plane perpendicular to [001], Adjacent nanoribbons are held together only by weak van der Waals forces. The bonding strength is significantly lower than that of covalent bonds, leading to weak electronic coupling and requiring carriers to overcome potential barriers for transport.

This fundamental difference of “strong intra-chain vs. weak inter-chain” bonding dictates the material’s strong anisotropy in electrical, optical, and defect behaviors, directly determining the performance quality of photovoltaic devices under different crystal orientations [51].

### Anisotropic physical mechanisms of carrier transport

**Fig. 2:**
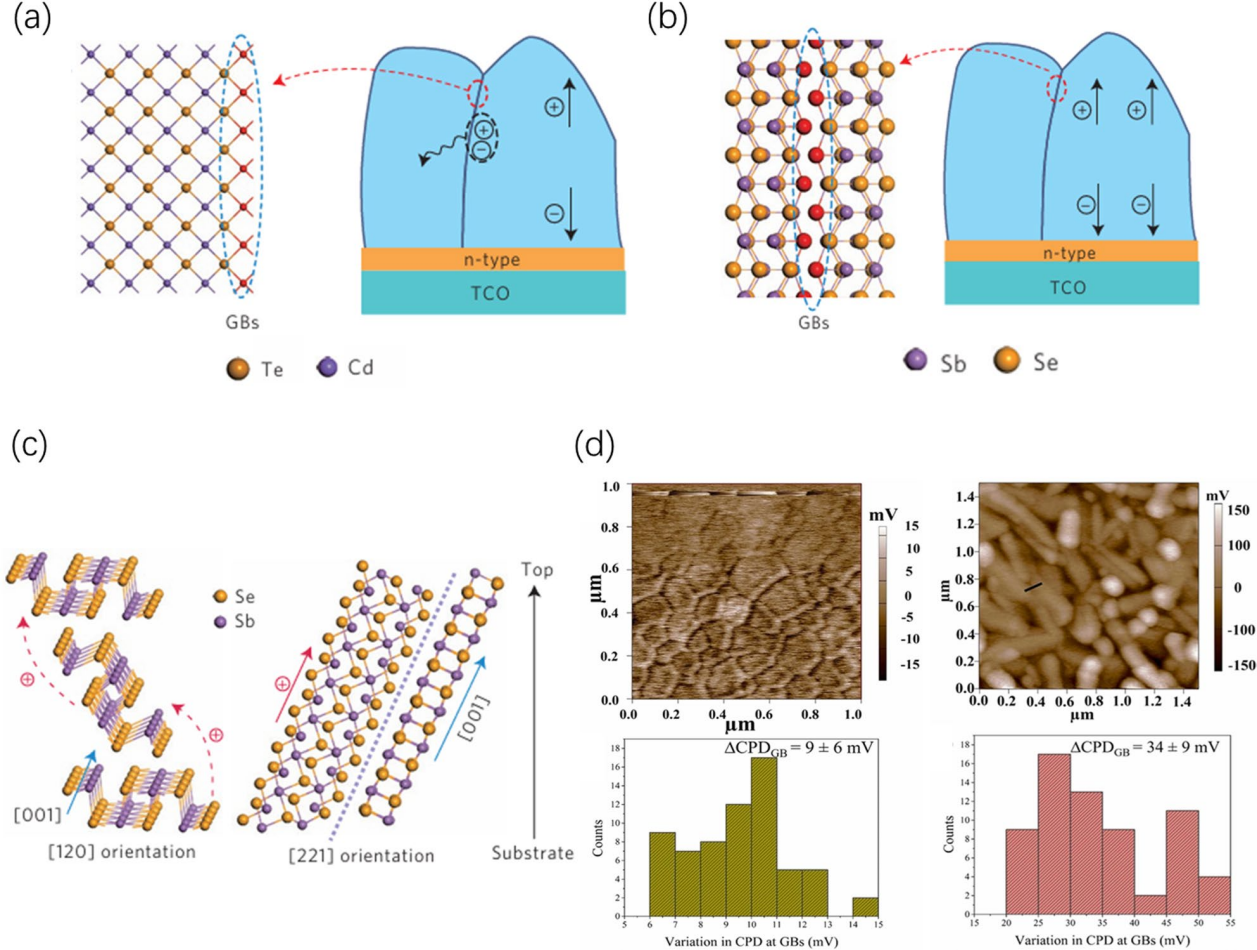
**a** CdTe possesses a 3D crystal structure; dangling bonds at its grain boundaries act as defects leading to recombination losses of photogenerated carriers. **b** “Benign grain boundaries” formed by Sb_2_Se_3_ with [001] vertical orientation, featuring no dangling bonds and self-passivation. **c** Schematic of atomic arrangements for different orientations (e.g., [120] vs. [221]), revealing efficient transport channels along the [001] intra-chain covalent bond direction. Reproduced with permission [23]. Copyright 2015, Springer Nature. **d** Kelvin Probe Force Microscopy (KPFM) and Contact Potential Difference (CPD) distribution maps. It demonstrates weak potential bending (< 20 mV) at [hk1] oriented grain boundaries, confirming their electrically benign characteristics. Reproduced with permission [58]. Copyright 2023, Elsevier B.V

Crystal orientation profoundly influences migration mechanisms and transport efficiency by defining the carrier transmission pathways, primarily manifesting in two modes (Fig. 2c):

Band Transport along Covalent Bonds: When Sb_2_Se_3_ or Sb_2_S_3_ thin films exhibit a [hk1] preferred orientation, photogenerated carriers are transported mainly along the direction of strong covalent bonds. Systematic characterization by Chen et al. demonstrated significantly higher carrier mobility in this direction. For instance, hole mobilities along the a- and b-axes were 1.17 and 0.69 cm^2^‧V^−1^‧s^−1^, respectively, whereas hole mobility along the c-axis reached approximately 2.59 cm^2^‧V^−1^‧s^−1^, consistent with a band conduction mechanism [52, 53]. This is attributed to strong orbital overlap, low effective electron mass, and extended wave functions along the covalent bond direction, allowing carriers to move freely within continuous band states [54].

Hopping Conduction across van der Waals Gaps: If the film possesses an [hk0] orientation where nanoribbons lie parallel to the substrate, carriers must traverse van der Waals gaps to transport across the film thickness^12^. Early electrical and magnetic studies on Sb_2_S_3_ single crystals indicated that these gaps act as potential barriers, interrupting the continuous energy bands. Consequently, carrier transport relies on a thermally activated hopping mechanism [55–57]. This process is accompanied by strong scattering and localization effects, leading to a drastic reduction in mobility and a significant increase in series resistance, which is detrimental to current collection in devices.

### Defect physics of “benign grain boundaries”

In polycrystalline thin-film solar cells, grain boundaries often serve as recombination centers due to defects such as dangling bonds, limiting the open-circuit voltage and fill factor (Fig. 2a). However, owing to their one-dimensional structural characteristics, antimony-based chalcogenides can form “benign grain boundaries” under specific orientations [23].

#### Absence of dangling bonds and grain boundary self-passivation

From a crystallographic perspective, crystal planes parallel to the [001] direction consist of the complete lateral surfaces of [Sb_4_X_6_]_n_ units. These surfaces are chemically saturated and do not generate dangling bonds. Therefore, when grains grow with [hk1] orientation and coalesce, the resulting grain boundaries are primarily composed of these saturated planes. Theoretically, this avoids the introduction of deep-level defect states, thereby achieving grain boundary self-passivation (Fig. 2b) [59, 60]. This feature enables [hk1]-oriented films to maintain high carrier lifetime and collection efficiency even with high grain boundary densities.

#### Surface potential and band bending

Recent nanoscale electrical characterizations have provided direct evidence for “benign grain boundaries”. Vashishtha et al. utilized Kelvin Probe Force Microscopy (KPFM) to compare the grain boundary potential distribution in Sb_2_Se_3_ films with different orientations. They found that the surface potential variation (ΔCPD) at [hk1]-oriented grain boundaries was typically less than 20 mV, indicating weak band bending and narrow space charge regions that carriers can traverse without hindrance. In contrast, non-[hk1] (e.g., [hk0]) oriented grain boundaries exhibited larger potential fluctuations reaching 40–50 mV, corresponding to strong band bending and higher defect charge densities, making them prone to becoming recombination centers (Fig. 2d) [58].

These results align with earlier KPFM and Electron Beam Induced Current (EBIC) characterizations by Zhou et al. (2015), further confirming the critical impact of orientation on grain boundary electronic activity. Additionally, Thermal Admittance Spectroscopy (TAS) analysis showed that [221]-oriented films possess lower defect densities dominated by shallow-level defects, consistent with their superior photovoltaic performance [52, 61].

### Correlation between orientation regulation and device performance

Synthesizing the physical mechanisms discussed above, crystal orientation plays a decisive role in antimony-based chalcogenide photovoltaic devices. It directly influences the power conversion efficiency through the synergistic effect of constructing high-speed carrier transport channels and suppressing non-radiative recombination at grain boundaries.

The mapping relationship from microscale to macroscale indicates that a [hk1] preferred orientation ensures [Sb_4_X_6_]_n_ nanoribbons are aligned perpendicular to the substrate. This eliminates the hindrance of interlayer van der Waals barriers to longitudinal carrier transport, substantially reducing series resistance to enhance the Fill Factor (FF). The “benign grain boundaries” associated with this orientation effectively reduce dangling bond density and suppress carrier recombination losses, thereby significantly improving the Open-Circuit Voltage (V_OC_) [62]. Furthermore, optimized vertical orientation is typically accompanied by a denser film morphology and longer carrier diffusion lengths, ensuring effective collection of photogenerated carriers before recombination, which also benefits the Short-Circuit Current Density (J_SC_). Conversely, [hk0] orientation becomes a major bottleneck for device performance due to hopping transport across van der Waals gaps and high defect activity at grain boundaries.

Extensive experimental research has confirmed that high-efficiency antimony-based solar cells consistently exhibit significant [hk1] textural features. Regulating crystal orientation has become a critical pathway to approaching the Shockley-Queisser theoretical limit [63]. Therefore, achieving precise control over crystal orientation through the various strategies discussed in subsequent sections is not only central to optimizing antimony-based photovoltaic performance but also provides important theoretical guidance and technical reference for orientation engineering in other low-dimensional semiconductor materials.

## Thermodynamic and kinetic competition mechanisms in thin film growth

### Surface energy minimization: the thermodynamic tendency for “lie-down” growth

The orientation selection of crystals on a substrate is primarily governed by the principle of thermodynamic minimization, meaning the system tends towards the state with the lowest Gibbs free energy. For Sb_2_X_3_ (X = S, Se) crystals with quasi-one-dimensional chain-like or ribbon-like structures, the surface energies of different crystal planes exhibit significant anisotropy. Crystal planes parallel to the axis of the strongly covalently bonded [Sb_4_X_6_]_n_ nanoribbons (such as (120), (020), (230), collectively referred to as (hk0) planes) involve the breaking of weak van der Waals interactions and thus possess the lowest surface energy (γ_hk0_). Conversely, (hk1) planes (such as (221), (211), (041)) that are perpendicular to or at an angle to the nanoribbon axis require the scission of stronger covalent bonds, resulting in higher surface energy (γ_hk1_ > γ_hk0_) [64].

From the perspective of thermodynamic equilibrium, to minimize the total surface energy of the system, crystal nuclei initially prefer to contact the substrate with their lowest-surface-energy (hk0) planes. This leads to an intrinsic tendency for [Sb_4_X_6_]_n_ nanoribbons to naturally “lie down” parallel to the substrate surface. This phenomenon has been verified in numerous experiments. For example, Pattini et al. found that on amorphous glass or molybdenum substrates, Sb_2_Se_3_ films exhibit a strong (hk0) preferred orientation over a wide temperature range (200–350 °C), with nanoribbons almost completely lying flat on the surface [65]. Similarly, Govindharajulu et al. observed that when preparing Sb_2_Se_3_ films via close-spaced sublimation, thermodynamically more stable (hk0) orientations (such as (230), (240)) gradually become dominant as the growth temperature rises above 275 °C [66]. Although this “lie-down” structure is thermodynamically stable, it hinders the transport of photogenerated carriers along the film thickness direction, becoming a significant factor limiting solar cell performance.

### Interface interactions and substrate effects in heterogeneous nucleation

**Fig. 3:**
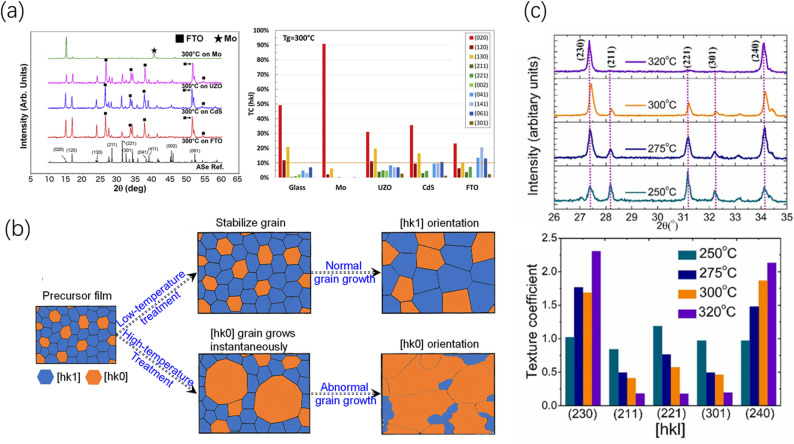
**a** XRD patterns and texture coefficients of Sb_2_Se_3_ on different substrates (Glass, Mo, CdS, FTO, UZO). Reproduced with permission [65]. Copyright 2020, Elsevier B.V. **b** Schematic of grain growth modes, illustrating the competition between “normal grain growth” (maintaining the nucleated [hk1] orientation) and “abnormal grain growth” (large [hk0] grains consuming small grains driven by thermodynamics). Reproduced with permission [67]. Copyright 2023, Royal Society of Chemistry. **c** Evolution of XRD patterns and texture coefficient comparison for Sb_2_Se_3_ films grown on glass substrates at different deposition temperatures. Reproduced with permission [66]. Copyright 2024, Elsevier B.V

To overcome the spontaneous formation of the (hk0) preferred orientation—which is detrimental to vertical charge transport—on amorphous glass or molybdenum (Mo) substrates, Pattini et al. explicitly pointed out that substrate selection is key to shifting crystal orientation from “lie-down” to “upright”. Their experiments demonstrated that by switching the deposition substrate to materials such as Fluorine-doped Tin Oxide (FTO), Cadmium Sulfide (CdS), or Undoped Zinc Oxide (UZO), one can actively and significantly intervene in the crystallization orientation of the film (Fig. 3a) [65].

This study provided direct experimental evidence: for Sb_2_Se_3_ films grown on FTO substrates, the proportion of non-conductive (hk0) oriented grains dropped drastically from 99.4% (on Mo) to 39.8%; simultaneously, (hk1) orientations with vertical conductive components (such as (041), (141), and (061)) were significantly enhanced. This transition was attributed to the template effect of the substrate, implying that the surfaces of these oxide/sulfide substrates likely formed more favorable interfacial interactions with specific crystal planes of Sb_2_Se_3_.

From the perspective of crystal growth mechanisms, these results reveal that the nucleation process is dominated by the substrate interface. When the substrate surface matches a specific (hk1) plane of Sb_2_Se_3_ chemically or lattice-wise, it creates a lower interfacial energy. This preferentially promotes the formation and growth of these “upright” oriented nuclei both thermodynamically and kinetically, ultimately achieving “vertical anchoring” of the nanoribbons. The work of Pattini et al. provided an empirical basis for the effectiveness of this interface engineering strategy.

Furthermore, introducing an ultrathin interface layer is also an effective strategy. For instance, Wu et al. introduced an approximately 1 nm thick ZnS interlayer on a SiO_2_ substrate. This not only increased the nucleation density of Sb_2_S_3_, yielding finer and more uniform grains, but also influenced competitive grain growth by improving interface binding energy. They observed a small amount of high-index oriented grains, such as [201] and [301], which may form high-mobility vertical transport channels [68].

### Grain growth kinetics: maintenance of metastable orientations and thermal relaxation

Following nucleation, the final orientation of the film deeply depends on the subsequent grain growth process, which serves as a stage for the competition between kinetic control and thermodynamic relaxation. Grain growth leads not only to an increase in average grain size but also to significant evolution in grain size distribution and orientation distribution, where stress, surface energy anisotropy, and spatial confinement effects play key regulatory roles.

#### Normal grain growth and kinetic “freezing”

At lower deposition temperatures or during rapid deposition, the surface diffusion capability of adatoms is limited, placing the system in a kinetically controlled regime. At this stage, the activation energy barriers required for lattice reconstruction or grain boundary migration are difficult to surmount. Consequently, the metastable [hk1] oriented structure formed during the heterogeneous nucleation phase (induced by the substrate) can be “frozen” and perpetuated during the subsequent growth process. This mode, where grains grow at approximately similar rates, is termed normal grain growth. Wu et al. clarified this phenomenon: subjecting Sb_2_S_3_ precursor films to low-temperature (e.g., starting from room temperature) slow-ramping annealing helps stabilize the initial [hk1] oriented nuclei. These nuclei then grow according to the normal grain growth mode during subsequent high-temperature annealing, ultimately yielding a film with [hk1] preferred orientation [67]. In this scenario, the film’s orientation is primarily determined by the nucleation process (substrate induction) rather than the thermodynamic equilibrium state.

#### Abnormal grain growth and thermodynamic “flip”

When sufficient thermal energy is provided—such as through direct high-temperature deposition or high-temperature rapid annealing—the system acquires enough atomic mobility to relax towards the global minimum free energy state (thermodynamic equilibrium). This triggers abnormal grain growth. During this process, grains with the lowest surface energy (i.e., the most stable [hk0] orientation) gain a tremendous thermodynamic driving force. Their grain boundary migration rate far exceeds that of other orientations. Through a mechanism similar to Ostwald ripening, these [hk0] grains rapidly consume the surrounding metastable [hk1] small grains and grow abnormally large (Fig. 3b). The work of Govindharajulu et al. intuitively demonstrated this “flip” process: when the growth temperature of Sb_2_Se_3_ was raised from 250 °C to 320 °C, the preferred orientation of the film transitioned from the [hk1]-direction (221) plane to the [hk0]-direction (230) plane (Fig. 3c) [66]. Wu et al. also observed that placing an Sb_2_S_3_ precursor film directly on a hot plate above 300 °C immediately induced abnormal grain growth, leading to the rapid formation and dominance of large [hk0] oriented grains [67].

In summary, the crystal orientation of Sb_2_X_3_ thin films is the result of a complex interplay between the thermodynamic minimization trend and kinetically controllable factors. Thermodynamic instinct drives nanoribbons to “lie down” to lower surface energy; however, by regulating heterogeneous nucleation and precisely controlling kinetic parameters such as temperature, rate, and growth environment, this equilibrium can be broken to induce and sustain metastable [hk1] oriented growth. Understanding and mastering this thermodynamic-kinetic competition mechanism is key to realizing high-performance anisotropic semiconductor thin-film devices.

## Optimization strategies for crystal orientation

### Solvent and precursor engineering

During the liquid-phase preparation of antimony-based chalcogenide thin films, the chemical environment of the solution is of paramount importance, directly shaping the nucleation dynamics and growth habits of the crystals. To overcome thermodynamic barriers and induce the formation of a vertical [hk1] orientation conducive to carrier transport, researchers have achieved refined customization of the entire crystallization process through precursor intervention strategies such as additive regulation, compositional optimization, and coordination engineering.

#### Additive regulation

Specific metal cations can achieve precise orientation guidance by intervening in the heterogeneous nucleation process. For instance, the introduction of Ag^+^ ions into the hydrothermal precursor solution, utilizing the ultrathin intermediate layer induced at the Mo substrate interface, can successfully steer Sb_2_Se_3_ from a random orientation mode to longitudinal [hk1] preferred growth, thereby constructing efficient photoelectric transport pathways [69]. Following a similar logic, the selective adsorption of the alkali metal ion Cs^+^ on the surface of nascent crystal nuclei can effectively suppress secondary nucleation, cultivating highly preferred-oriented, large-grain thin films through the Ostwald ripening mode, which demonstrates the universal significance of ion-mediated optimization in crystallization dynamics [70].

**Fig. 4:**
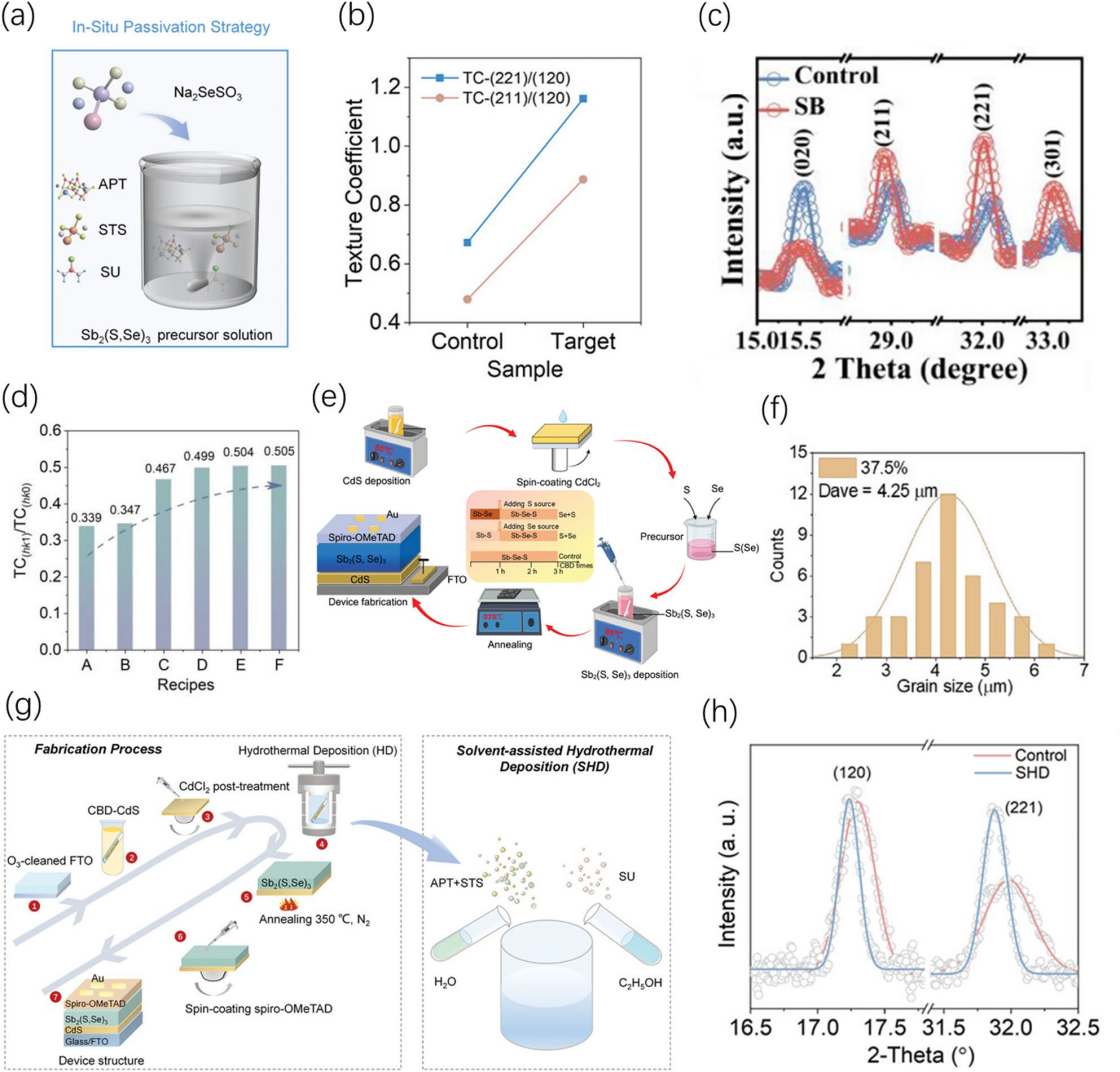
Schematic illustrations and results of solvent and precursor engineering strategies. **a** Schematic illustration of the in situ Na_2_SeSO_3_ passivation strategy. **b** Texture coefficient ratios of the [221] and [211] planes relative to the [120] plane for Sb_2_(S,Se)_3_ thin films following in situ Na_2_SeSO_3_ passivation. Reproduced with permission [31]. Copyright 2024, Wiley–VCH GmbH. **c** Magnified XRD patterns of Sb_2_(S,Se)_3_ thin films with and without the addition of SB. Reproduced with permission [72]. Copyright 2025, Wiley–VCH GmbH. **d** TC ratios between the (hk1) and (hk0) crystalline planes of the optimal Sb_2_S_3_ films based on recipes A–F. Reproduced with permission [18]. Copyright 2024, Wiley–VCH GmbH. **e** Flowchart of the solar cell fabrication process using a sequential Se + S deposition strategy. The Se-rich environment promotes Se 4p–Sb 5p orbital hybridization and vertical growth. Reproduced with permission [73]. Copyright 2024, Wiley–VCH GmbH. **f** Statistical histogram of the average grain size for Sb_2_S_3_ thin films prepared via the 37.5% S-CBD method. Reproduced with permission [74]. Copyright 2024, Wiley–VCH GmbH. **g** Solvent-assisted hydrothermal deposition (SHD) strategy. Ethanol is utilized as a partial solvent during the deposition process. **h** Magnified XRD patterns of the corresponding Sb_2_(S,Se)_3_ thin films. Reproduced with permission [75]. Copyright 2023, Wiley–VCH GmbH

Furthermore, additives act not only as orientation inducers but also play a role in optimizing electrical properties. Chen et al. adopted a Cl and Se co-doping strategy in the Sb_2_S_3_ system, which not only filled intrinsic defects such as sulfur vacancies but, more importantly, effectively induced the controlled growth of grains along the vertical direction, significantly enhancing carrier mobility and device efficiency [71]. The in-situ passivation strategy targeting the initial nucleation stage represents the current refined development direction of additive engineering; Zhao et al. introduced Na_2_SeSO_3_ as an additive in the solution to rapidly release Se^2−^ in the early stage of the reaction to promote rapid grain nucleation (Fig. 4a). This “speed over disorder” approach effectively suppressed the generation of grain boundary defects and induced a favorable [hk1] orientation (Fig. 4b), pushing the device efficiency beyond 10.81% [31]. Additionally, Fu et al. recently achieved a “two birds with one stone” effect by introducing sodium borohydride (SB): on one hand, the alkaline environment provided by SB accelerated the decomposition of the selenium source and the nucleation of Sb_2_Se_3_, synergistically eliminating deep-level Sb_Se_ defects; on the other hand, its strong reducing property was utilized to reduce SbO^+^ in the solution to Sb^3+^, suppressing the formation of the insulating impurity Sb_2_O_3_ from the source. This induced a high-purity thin film with a significant [hk1] preferred orientation (Fig. 4c), driving device efficiency to 10.62% [72]. These additive engineering approaches can artificially intervene in the chemical environment during the initial nucleation stage, breaking the transverse growth inertia of the quasi-one-dimensional segments of antimony-based materials and transforming them into vertical structures conducive to longitudinal charge extraction, which has become a crucial pathway for enhancing device performance.

#### Composition and solvent optimization

Composition engineering primarily finely intervenes in nucleation and growth pathways from the chemical potential level by regulating precursor activity. Research has found that adjusting the Se/S ratio in the hydrothermal system not only achieves a tunable bandgap but also thermodynamically promotes the preferred rearrangement of [Sb_4_(S,Se)_6_]_n_ segments. The multi-sulfur source synergistic strategy developed by Wang et al. utilizes the difference in hydrolysis and complexation rates between sodium thiosulfate (STS) and thioacetamide (TAA) to effectively balance the nucleation and growth rates, significantly improving crystallization quality. Furthermore, the crystal orientation was effectively tailored by employing various sulfur source formulations (Fig. 4d) [18]. Subsequently, Dai et al. proposed a sequential chemical bath deposition strategy, enhancing orbital hybridization by first depositing a Se-rich layer and then introducing a sulfur source (Fig. 4e). This successfully induced a crystal orientation favorable for longitudinal transport, yielding a device efficiency of 9.29%, which was the highest value for the chemical bath deposition method at that time [73].

The introduction of co-solvents into the solvent can alter the physicochemical properties of the solution environment, achieving profound control over grain orientation. Introducing ethanol into the hydrothermal reaction (SHD strategy) (Fig. 4g) can significantly enhance the preferred orientation of the thin film along the (221) crystal plane (Fig. 4h) by reducing precursor supersaturation and delaying ion release, helping the efficiency surpass 10.75% [75]. Utilizing ethanol assistance in chemical bath deposition (S-CBD) can induce the growth of large grains up to 4.25 μm in size by adjusting solution polarity and supersaturation (Fig. 4f) [74]. The core of this kinetic regulation logic lies in suppressing random nucleation during subsequent growth through precise external environmental intervention, maintaining a highly ordered vertical structure. Overall, composition optimization provides the “internal driving force” to regulate reactant activity, while solvent engineering constructs the “external environment” to control the crystallization rate.

#### Chelating coordination effect

**Fig. 5:**
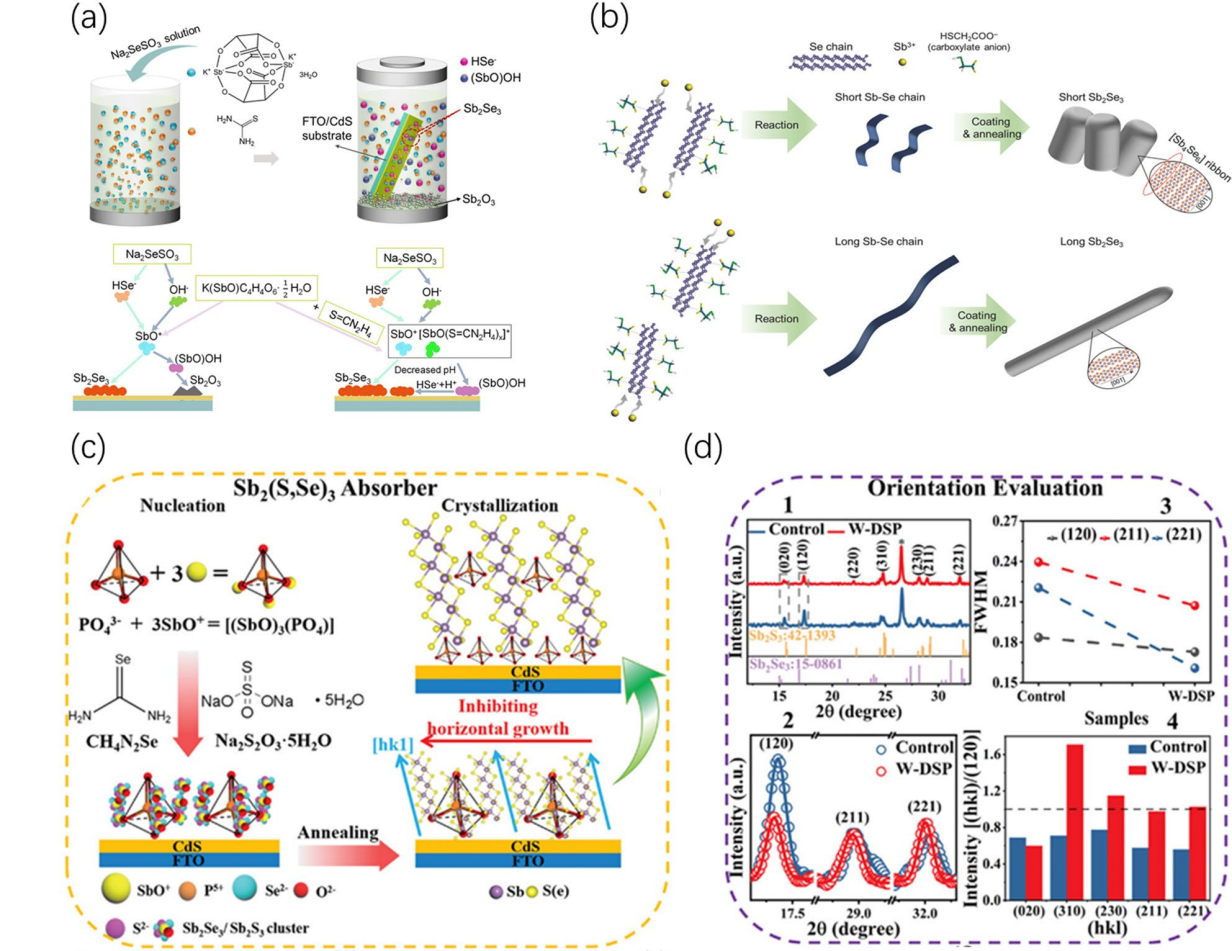
**a** Deposition mechanism of Sb_2_Se_3_ thin films. Reproduced with permission [76]. Copyright 2021, American Chemical Society. **b** Schematic illustration of the formation mechanism of Sb_2_Se_3_ nanostructures. A few carboxylate anions partially adsorb on the Se chains and Sb^3+^ react with the Se chains, resulting in the formation of short Sb_2_Se_3_ nanostructures. Abundant carboxylate anions fully adsorb on the Se chain and the lateral growth of Sb_2_Se_3_ is restricted by these ions, consequently leading to the formation of long Sb_2_Se_3_ nanostructures. Reproduced with permission [77]. Copyright 2018, Wiley–VCH GmbH. **c** Schematic illustration of PO_4_^3−^-assisted growth of Sb₂(S,Se)₃ thin films, demonstrating that PO_4_^3−^ and SbO^+^ form coordination clusters to delay Sb^3+^ release, suppress transverse growth of nanoribbons, and induce [hk1] vertical orientation. **d** Comparison of experimental data for Sb_2_(S,Se)_3_ film orientation with and without DSP: 1) XRD patterns; 2) Magnified XRD patterns; 3) FWHM values of (120), (211), and (221) diffraction peaks; 4) Texture coefficients. Reproduced with permission [78]. Copyright 2024, Wiley–VCH GmbH

The essence of the chelating coordination effect lies in the formation of stable complexes through electron cloud overlap between ligands and precursor metal ions (e.g., Sb^3+^), thereby reshaping the existence state of the precursors at the molecular scale. This effect is primarily manifested as a kinetic modification of the nucleation process, achieving a “deceleration” effect by regulating the ion release rate. For example, introducing the strong chelating agent EDTA can utilize its multidentate coordination capability to disperse precursor aggregates, transforming the deposition mode from random stacking to a controlled ionic reaction [29]; thiourea (TU), on the other hand, reduces solution supersaturation through complexation, inducing nanoribbons to stack regularly along the vertical direction, The schematic diagram of its deposition is shown in Fig. 5a [76]. In addition, introducing organic ligands in different proportions into the precursor solution, such as thioglycolic acid and ethanolamine, can precisely regulate the anisotropic growth of Sb_2_Se_3_ one-dimensional nanostructures; by controlling the proportion of coordination molecules to alter the aspect ratio of nanorods, the formation of highly preferred-oriented arrays is induced (Fig. 5b) [77]. These precursor clusters can adsorb in a vertically aligned manner conducive to charge transport, ultimately achieving a fundamental transition of the thin film from a random distribution to a controlled orientation. As research deepens, regulation strategies have evolved to utilize the geometric steric hindrance and interfacial energy manipulation of coordination molecules. Ren et al. recently developed a PO_4_^3−^ tetrahedron-assisted strategy (Fig. 5c), where by forming a [(SbO)_3_(PO_4_)] complex with Sb^3+^, they utilized significant steric hindrance kinetics to suppress the transverse growth of nanoribbons, forcing grains to nucleate along the [hk1] direction and grow vertically (Fig. 5d), ultimately achieving a photoelectric conversion efficiency of 10.67% [78].

### Regulation of deposition process parameters

The physical vapor deposition process essentially constructs an energy field that highly deviates from the equilibrium state, and the selection of crystal orientation depends on the competition between the thermodynamically stable state and the kinetic driving force. Regulating process parameters based on vacuum or vapor deposition technologies is the core approach to achieving the transition of Sb_2_(S,Se)_3_ thin films from thermodynamically favorable transverse growth to kinetically favorable vertical orientation. By precisely controlling the evaporation source temperature [79], substrate temperature, deposition rate, and vapor dynamics, researchers can directly intervene in the energy supply and mass transfer during the nucleation and growth processes, fundamentally determining the microstructure and photoelectric properties of the thin films.

#### Temperature and thermal field regulation

Because the unique quasi-one-dimensional chain-like structure of Sb_2_(S,Se)_3_ is formed by [Sb_4_X_6_]_n_ (X = S, Se) units bonded by strong covalent bonds and stacked via van der Waals forces, its growth process exhibits strong anisotropy. The pioneering work of Zhou et al. established the understanding of thermal field regulation in this field; they discovered that utilizing the rapid thermal evaporation (RTE) process can precisely control the thermal field gradient, inducing one-dimensional ribbons to grow perpendicularly to the substrate [23]. This directional growth mode effectively circumvents the recombination centers at the grain boundaries of traditional three-dimensional crystals, constructing a “highway” for carrier transport, and verified the significance of vertical orientation in passivating benign grain boundaries in early studies.

**Fig. 6:**
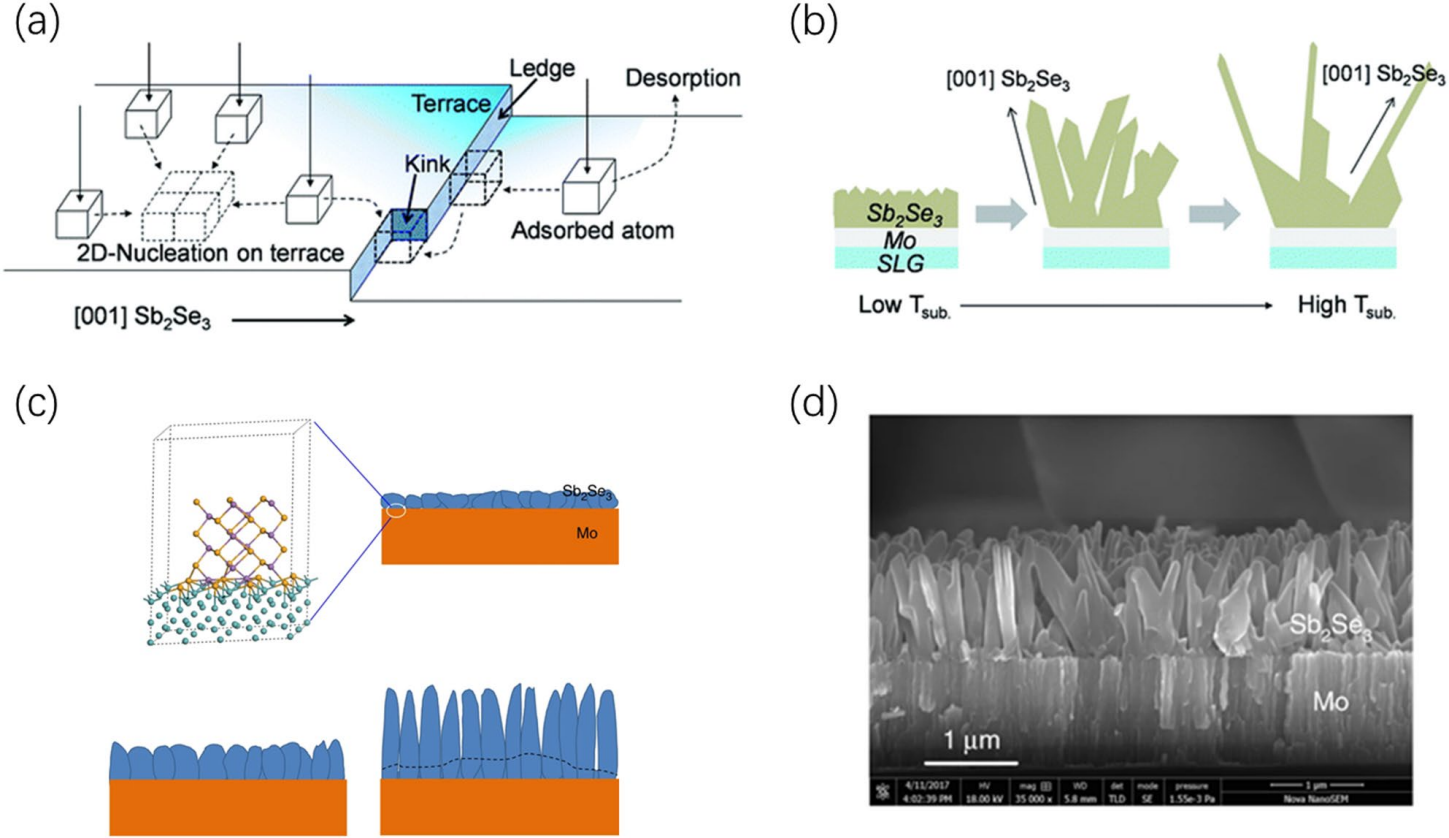
**a** Terrace-ledge-kink (TLK) model for Sb_2_Se_3_ grain growth; in this simplified TLK model, a cube represents an atom. **b** Substrate temperature-dependent morphological changes of Sb_2_Se_3_ thin films. Reproduced with permission [80]. Copyright 2019, Royal Society of Chemistry. **c** Growth model of Sb_2_Se_3_ nanorod arrays on Mo substrates, showing schematic illustrations at different growth stages. **d** Cross-sectional SEM image of Sb_2_Se_3_ nanorod arrays. Reproduced with permission [11]. Copyright 2019, Springer Nature

Building on this thermodynamic foundation, Park et al. further revealed the complexity of orientation evolution from a kinetic perspective, pointing out that the transition of Sb_2_Se_3_ from a flat thin film to a nanorod array is subject to an extremely narrow substrate temperature window. Based on the “terrace-ledge-kink (TLK)” model (Fig. 6a), temperature determines the energy distribution of nucleation sites on different crystal planes by regulating the diffusion coefficient of precursor atoms on the substrate surface. Only within a specific kinetic regime can the thermodynamically stable [hk0] plane and the kinetically favorable [hk1] direction be balanced, thereby achieving a highly preferred vertical array structure, The effect of substrate temperature on the morphology of Sb_2_Se_3_ thin films is shown in Fig. 6b [80]. Li et al. successfully prepared pure-phase Sb_2_Se_3_ nanorod arrays highly aligned along the [001] direction by optimizing the thermal field-assisted process (Fig. 6c), and the cross-sectional SEM image in Fig. 6d demonstrates the ordered orientation of Sb_2_Se_3_, significantly reducing non-radiative recombination losses and optimizing charge collection pathways [11]. This strongly demonstrates that the substrate temperature is not merely a variable for adjusting grain size but the decisive switch for breaking the thermodynamic limitations of one-dimensional structures and inducing orientation reconstruction.

#### Vapor deposition and kinetic regulation

**Fig. 7:**
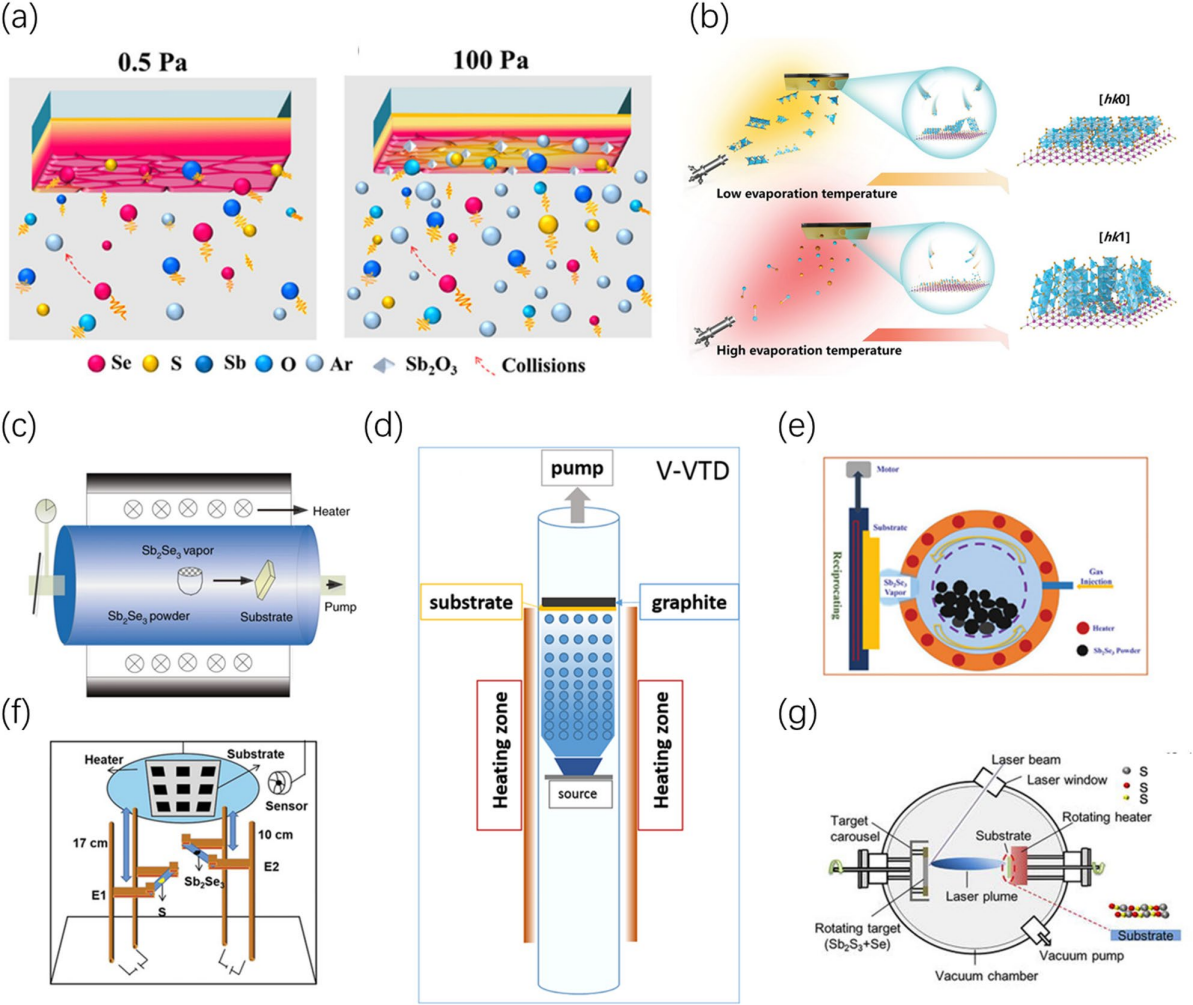
Schematic illustration of the regulation of various parameters during the vapor deposition process and different deposition systems. **a** Schematic illustrations of Sb_2_(S,Se)_3_ thin films deposited under different pressures in vapor transport deposition (VTD). Reproduced with permission [81]. Copyright 2025, Wiley–VCH GmbH. **b** Schematic illustration of the growth mechanism of Sb_2_Se_3_ thin films dependent on evaporation temperature in molecular beam epitaxy (MBE). Reproduced with permission [82]. Copyright 2024, Wiley–VCH GmbH. **c** Schematic of the VTD system. Reproduced with permission [24]. Copyright 2018, Springer Nature. **d** Schematic of the V-VTD deposition system. Reproduced with permission [83]. Copyright 2020, John Wiley & Sons, Ltd. **e** Schematic of the injection vapor deposition system. Reproduced with permission [25]. Copyright 2022, Wiley–VCH GmbH. **f** Schematic of the co-evaporation system, where the source-to-substrate distance and temperature for the two sources can be adjusted independently. Reproduced with permission [84]. Copyright 2021, Wiley–VCH GmbH. **g** Schematic illustration of the pulsed laser deposition (PLD) apparatus. Reproduced with permission [85]. Copyright 2020, AIP Publishing

During the vapor deposition process, by adjusting the vacuum pressure and precursor vapor flow rate, the nucleation and growth pathways of Sb_2_(S,Se)_3_ can be precisely intervened from the perspective of molecular dynamics. By regulating the mean free path (MFP) of vapor molecules and the precursor decomposition energy levels, the thermodynamic equilibrium is broken to achieve the [hk1] preferred orientation. The mean free path modulation (MFPM) strategy proposed by Wang et al. demonstrates that at lower pressures (e.g., 0.5 Pa), the molecular MFP significantly increases, and the “evolutionary selection” growth mode of high-kinetic-energy molecules is enhanced (Fig. 7a), thereby inducing the thin film to grow in a highly ordered manner along the [hk1] direction [81]. Furthermore, the decomposition degree of precursor molecules directly affects crystallization quality; Li et al. utilized molecular beam epitaxy (MBE) technology to reveal the intrinsic logic between evaporation source energy driving and orientation, finding that under a specific energy level window, the [hk1] crystal plane dominates the competition due to its lower growth energy barrier (Fig. 7b) [82].

Addressing the regulatory mechanism of growth kinetics on crystal orientation during vapor deposition, Wen et al. utilized vapor transport deposition (VTD) technology to improve thin film crystallinity through gas flow driving and pressure regulation, reducing deep-level defect density by an order of magnitude and proving the significant role of kinetic optimization in enhancing carrier transport [24]. Subsequently, Zhang et al. pioneered the verification of a “deposition-reevaporation” competition model in a vertical vapor transport deposition (V-VTD) system; by finely adjusting the thermodynamic parameters in the flow field, they successfully achieved the controllable switching of Sb_2_S_3_ thin film orientation from [hk0] to [hk1] [83]. Building on this, to further overcome the defect of unstable vapor flow in traditional sublimation methods, Duan et al. developed injection vapor deposition (IVD) technology, constructing a continuous supersaturation environment on the substrate surface through precise gas injection, which induced highly dense Sb_2_Se_3_ thin films with a strong [00 l] preferred orientation, thereby significantly improving device performance [25]. Yin et al. revealed the manipulative effect of vapor introduction timing on interfacial reaction kinetics through a multi-source sequential co-evaporation device; by optimizing the collision and reaction sequence between components, an efficiency breakthrough of 8.0% was achieved in vapor-deposited Sb_2_(S,Se)_3_ cells [84]. These studies collectively indicate that the essence of pressure regulation is the balance between kinetic energy and collision frequency; by endowing deposited particles with sufficient surface mobility, they can overcome orientation energy barriers and align efficiently along the vertical direction [86].

#### Sputtering and non-equilibrium deposition

By utilizing physical methods such as magnetron sputtering or pulsed laser deposition (PLD), deposited particles can acquire instantaneous kinetic energy far exceeding that of traditional thermal evaporation processes. This non-equilibrium deposition process, by conferring extremely high surface mobility to atoms, can overcome thermodynamic barriers and achieve precise regulation of the alignment direction of quasi-one-dimensional segments. The working pressure during the sputtering process is the core variable determining the energy distribution of deposited particles; Liang et al.’s research found that in a low-pressure environment, high-kinetic-energy atoms tend to form a thermodynamically stable [hk0] orientation, whereas when the pressure increases, the increased collision frequency alters the kinetic energy spectrum of the particles, inducing the thin film to grow with an inclination towards the [hk1] direction. This pressure-driven orientation transition not only optimizes the microstructure of the thin film but also significantly enhances its conductivity [87].

PLD technology utilizes extremely high-energy–density laser pulses to generate a highly active, highly ionized plasma plume. Under the non-equilibrium growth environment of PLD, the high-energy particle stream endows deposited atoms with extremely strong surface migration capabilities, enabling them to overcome the limitations of van der Waals stacking and spontaneously select crystal planes conducive to longitudinal carrier transport for growth. By precisely controlling the plasma kinetic process, researchers obtained high-quality absorber layers with highly preferred orientations, ultimately achieving a conversion efficiency of 7.05% in the corresponding devices [85]. Compared to chemical methods relying on thermal equilibrium diffusion, “energy-driven” processes like sputtering and PLD possess stronger process robustness and can maintain high crystallization quality at lower substrate temperatures. This non-equilibrium deposition not only induces the ideal vertical structure but also effectively reduces bulk defects and interfacial energy barriers by enhancing atomic exchange at the interface.

### Interface and substrate engineering

In addition to the precursor state, the physicochemical properties of the substrate surface also play a key role in determining the initial nucleation thermodynamics and subsequent growth kinetics of the thin films. Because Sb_2_(S,Se)_3_ has a unique quasi-one-dimensional crystal structure, its [Sb_4_(S,Se)_6_]_n_ covalent chains tend to grow parallel to the substrate driven by thermodynamics, forming a [hk0] orientation that hinders carrier transport. To break through this inherent thermodynamic limitation and induce a vertical [hk1] orientation conducive to charge extraction, researchers have developed multi-dimensional interface engineering strategies, encompassing a comprehensive intervention system from atomic-scale lattice matching to microscopic morphology modification.

#### Lattice matching and heteroepitaxial growth

**Fig. 8:**
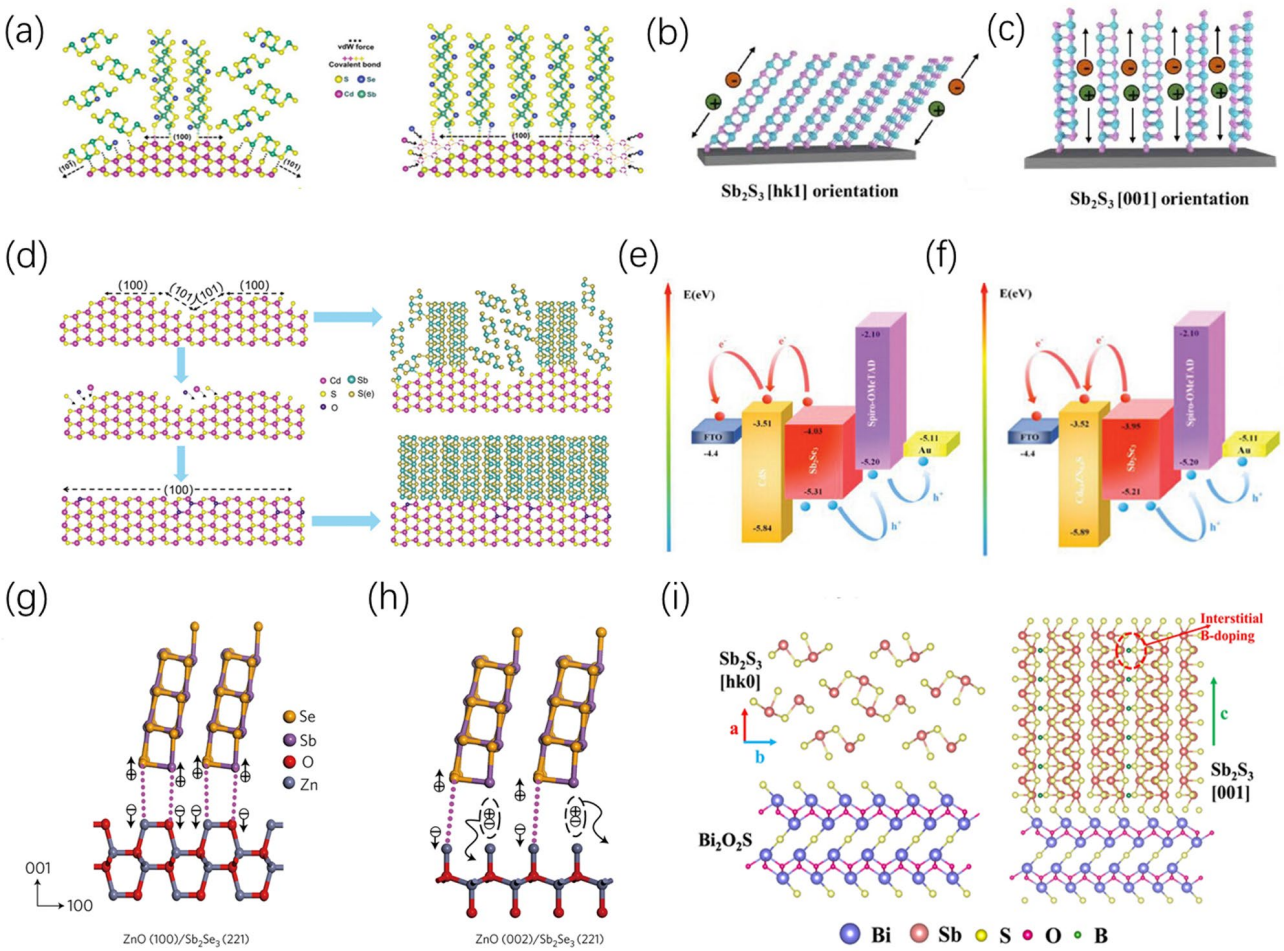
**a** Schematic demonstration of two different growth characteristics of Sb_2_(S,Se)_3_ on the (100) and (101) crystal planes of CdS (the (100) plane favors vertical anchoring, while the (101) plane favors horizontal lying); with the aid of extra Cd^2+^ in the hydrothermal system, the vertical growth of Sb_2_(S,Se)_3_ thin films on the CdS surface is achieved. Reproduced with permission [88]. Copyright 2021, Wiley–VCH GmbH. Schematic illustrations of the Sb_2_S_3_ thin films and charge transport for the **b** [hk1] and **c** [001] orientations. Reproduced with permission [89]. Copyright 2024, Elsevier. **d** Crystal growth model for the transformation of exposed CdS crystal planes induced by O-doped CdS thin films (left part), and the effect of CdS thin films with and without O doping on the crystallographic orientation of subsequently deposited Sb_2_(S,Se)_3_ (right part). Reproduced with permission [91]. Copyright 2023, Wiley–VCH GmbH. Schematic energy band structures of Sb_2_Se_3_ thin films **e** without and **f** with a Cd_0.6_Zn_0.4_S layer. Reproduced with permission [92]. Copyright 2024, Wiley–VCH GmbH. Atomic models of the [221]-oriented Sb_2_Se_3_ thin film on the **g** ZnO (100) plane and **h** ZnO (002) plane. Reproduced with permission [93]. Copyright 2017, Springer Nature. **i** Schematic illustration of the growth of (Sb_4_S_6_)_n_ nanoribbons on Bi_2_O_2_S nanosheet substrates, with interstitial boron atoms located between the nanoribbons. Reproduced with permission [94]. Copyright 2026, Elsevier

To overcome the growth anisotropy of the Sb_2_(S,Se)_3_ quasi-one-dimensional structure, heteroepitaxial technology is regarded as a fundamental means to induce the vertical alignment of crystals. Its essence lies in utilizing the lattice matching degree, chemical bonding, or surface energy characteristics between the buffer layer and the absorber layer to break thermodynamic limitations. Traditional sulfide buffer layers exhibit natural epitaxial advantages due to their similar chemical environment to antimony-based thin films. By precisely regulating the exposed crystal planes of the CdS buffer layer in the hydrothermal method, the solution-phase vertical epitaxial growth of Sb_2_(S,Se)_3_ on hexagonal CdS can be achieved, thereby significantly improving photoelectric conversion efficiency. Jin et al. utilized this method, introducing Cd^2+^ additives during the hydrothermal process to selectively consume the high-surface-energy (101) plane of CdS, thereby exposing more of the low-energy (100) plane, and successfully achieved the vertical anchoring of (Sb_4_X_6_)_n_ ribbon-like chains (Fig. 8a), pushing the device efficiency to 9.2% [88]. A similar mechanism is also reflected in vapor transport deposition (VTD) technology, where utilizing the [hk1] preferred-orientation nanorods successfully epitaxially grown on the In_2_Se_3_ surface can construct efficient charge transport channels (Fig. 8b,c) [89]. Research has further demonstrated that selecting sulfide substrates with lower lattice mismatch rates can effectively utilize the microscopic atomic arrangement at the interface to induce kinetically preferred growth of the absorber layer [90].

Beyond direct geometric matching, significant new progress has been made in recent years, with many researchers strengthening the inductive effect by compositionally modifying the buffer layer. For example, the in-situ oxygen-doped CdS thin film prepared by molecular beam epitaxy (MBE) technology can alter the growth habit of CdS to expose specific crystal planes, serving as an effective template to significantly enhance the [hk1] orientation of the subsequent Sb_2_(S,Se)_3_ (Fig. 8d) [91]. The diffusion of cation doping, such as Zn, in CdS not only optimizes interfacial band alignment (Fig. 8e, f) but also promotes the formation of a denser, larger-grained vertical structure in the absorber layer [92]. Even on oxide substrates, the epitaxial mechanism still plays a critical role. For instance, ZnO formed by spray pyrolysis [93] or exposed crystal planes of TiO_2_ regulated through thermal treatment [95] can both utilize oxygen vacancies as bridging sites to suppress interfacial defects and induce vertical orientation, thereby optimizing carrier transport. (Fig. 8g, h) Furthermore, utilizing the van der Waals epitaxial characteristics of two-dimensional (2D) materials such as Bi_2_O_2_S nanosheets [94] (Fig. 8i) and SnSe_2_[96] can significantly reduce interfacial stress and enhance carrier mobility. This epitaxial strategy has evolved into a comprehensive system combining chemical bonding, band engineering, and dimensional control, achieving precise regulation of the thin film’s microstructure.

#### Seed layer-assisted induced growth strategy

**Fig. 9:**
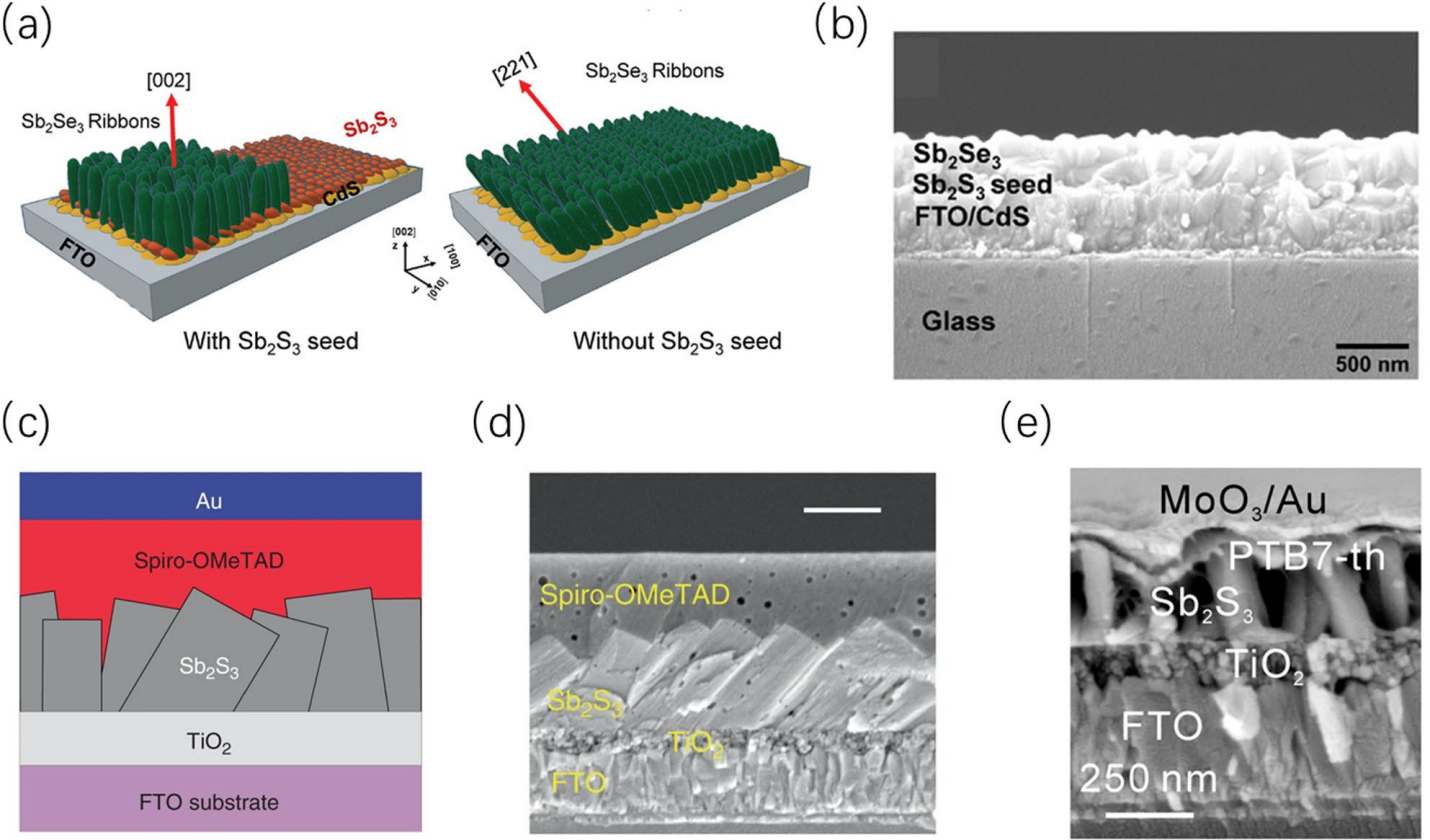
**a** Schematic illustration of the growth of Sb_2_Se_3_ nanoribbons on Sb_2_S_3_ seed layers and CdS layers. **b** Cross-sectional morphology of a device sample with a seed layer. Reproduced with permission [97]. Copyright 2022, Wiley–VCH GmbH. **c** Device structure of Sb_2_S_3_/ TiO_2_-BnPHJ. **d** Cross-sectional SEM characterization image of the device. Reproduced with permission [99]. Copyright 2019, Springer Nature. **e** Cross-sectional SEM characterization image of the p-i-n cell. Reproduced with permission [100]. Copyright 2021, Springer Nature

By utilizing a pre-grown seed layer, researchers can artificially construct a “structural template” to induce the subsequent thin film to undergo quasi-epitaxial growth along a specific [hk1] crystallographic axis, thereby breaking growth anisotropy at the initial nucleation stage. Because Sb_2_S_3_ and Sb_2_Se_3_ share the same space group and highly compatible lattice parameters, an ultrathin Sb_2_S_3_ seed layer is often used as a template to induce the growth of vertical nanoribbons (Fig. 9a, and the cross-sectional SEM image is shown in Fig. 9b). This not only improves charge transport along the direction of the quasi-one-dimensional segments but also significantly enhances device stability [97]. In substrate-configuration devices, introducing a controlled Sb_2_Se_3_ seed layer on a Mo substrate via close-space sublimation (CSS) can similarly induce the formation of a highly columnar and vertically oriented absorber layer, effectively passivating interfacial recombination losses [98].

Besides providing a structural template, the seed layer can also finely tune thin film morphology by altering nucleation kinetics. The in-situ growth of preferentially oriented single-crystalline cuboids [99] (The device structure and cross-sectional SEM image are shown in Fig. 9c and d) or nanorod arrays [100] (The cross-sectional SEM image of the device is shown in Fig. 9e) on polycrystalline TiO_2_ reveals the physical mechanism of “directional competitive epitaxial nucleation and growth”. Under this mechanism, tiny seeds act as nucleation centers to suppress disordered nucleation, allowing grains to undergo spatial confinement and orientation competition during the growth process, ultimately forming a vertical structure conducive to longitudinal carrier extraction. In fully vacuum-processed devices, the seed layer strategy also exhibits excellent process compatibility. Compared to traditional chemical bath deposition, the highly crystalline vacuum-processed seed layer can more effectively induce the absorber layer to exhibit a significant [221] orientation and construct a superior heterojunction interface [64]. In brief, the seed layer-assisted strategy has evolved into a multifunctional approach encompassing nucleation kinetic control and interfacial passivation [101], transforming the material’s natural growth anisotropy into the longitudinal electrical transport advantages required for devices by artificially intervening in the initial nucleation stage.

#### Interface engineering and chemical modification

**Fig. 10:**
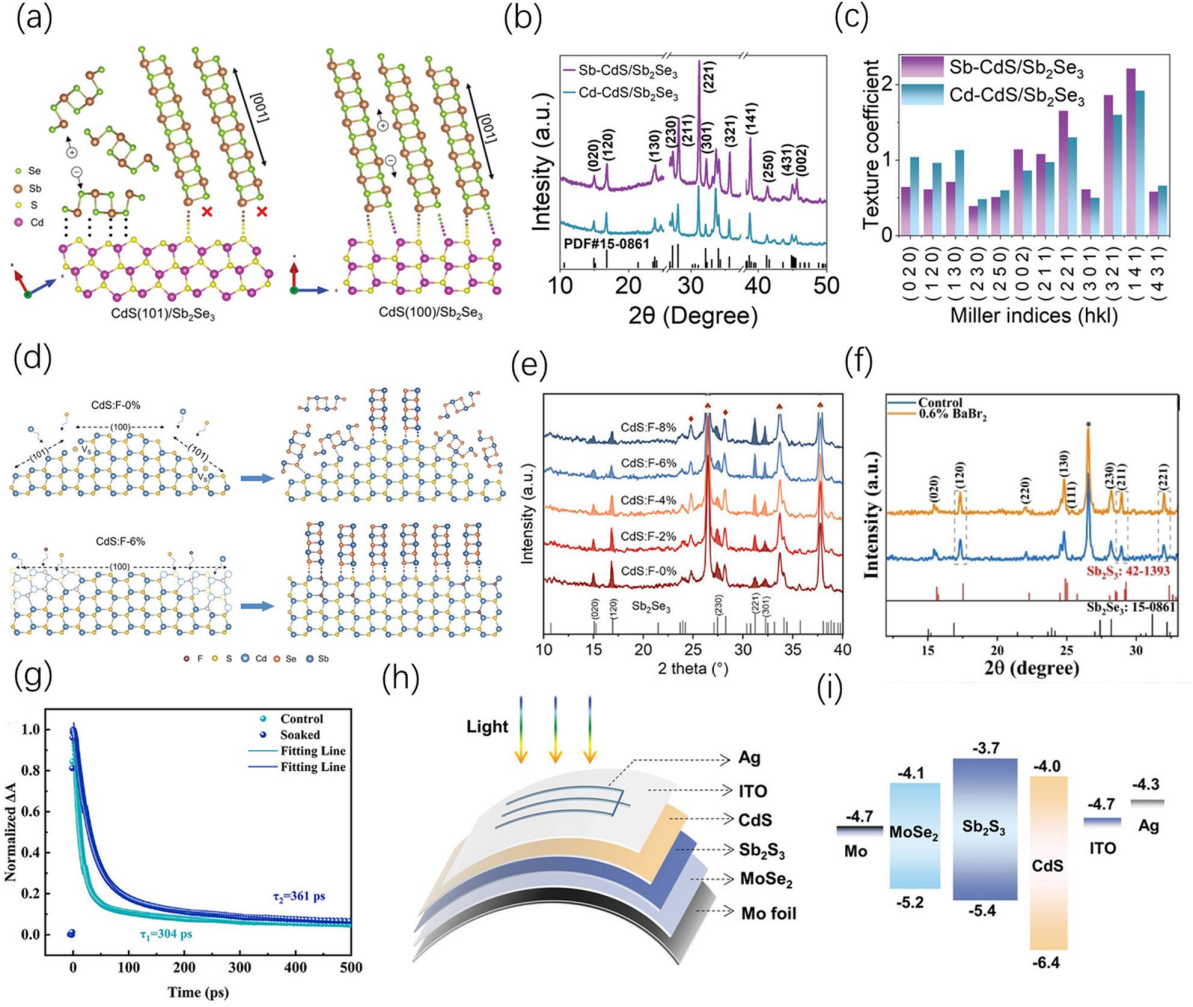
**a** Schematic illustration of the growth characteristics of (Sb_4_Se_6_)_n_ ribbons of Sb_2_Se_3_ on the (100) and (101) planes of CdS. **b** XRD patterns of Sb-CdS/Sb_2_Se_3_ and Cd-CdS/Sb_2_Se_3_ thin films. **c** Corresponding calculated texture coefficients. Reproduced with permission [102]. Copyright 2022, Wiley–VCH GmbH. **d** Crystal growth model of the change in exposed CdS crystal planes induced by F^−^-doped CdS thin films and its effect on the crystal orientation of subsequently deposited Sb_2_Se_3_. **e** XRD patterns of Sb_2_Se_3_ thin films deposited on F^−^-doped CdS with different concentrations (concentrations from 0 to 8%, with the FTO peaks marked by ♠). Reproduced with permission [103]. Copyright 2025, Wiley–VCH GmbH. **f** XRD patterns of Sb_2_(S,Se)_3_ thin films after control and 0.6% BaBr_2_ treatments. Reproduced with permission [104]. Copyright 2024, Wiley–VCH GmbH. **g** Carrier decay curves for the control and immersion-treated CdS samples. Reproduced with permission [105]. Copyright 2025, Wiley–VCH GmbH. **h** Device structure of the flexible substrate-based Sb_2_S_3_ solar cell. **i** Schematic energy band diagram of the respective layers. Reproduced with permission [106]. Copyright 2023, Wiley–VCH GmbH

Modifying the surface of the buffer layer or back electrode via chemical means can regulate the chemical potential and nucleation driving force at the interface, enabling the [hk1] crystal orientation to gain a kinetic advantage right from the nucleation stage, while synergistically resolving issues of interfacial recombination and band misalignment. In device fabrication, surface modification of the CdS buffer layer is particularly crucial. Cai et al. proposed using SbCl_3_ to treat the surface of CBD-CdS; combined with a post-annealing process, this not only induced the transformation of CdS into a stable hexagonal phase but also significantly improved surface smoothness, providing a better template for subsequent absorber layer growth (Fig. 10a). The XRD patterns of the Sb_2_Se_3_ thin films and the corresponding calculated texture coefficients are presented in Fig. 10b and c, respectively [102]. In addition, Shen et al. regulated the surface energy states of CdS through fluorine (F) doping, which can guide it to expose non-polar planes, thereby inducing the absorber layer to preferentially grow along the high-mobility direction (Fig. 10d), and the XRD pattern in Fig. 10e shows a significant enhancement in the [hk1] crystal orientation [103]. Ren et al. introduced BaBr_2_, utilizing the selective adsorption of Br^−^ ions on polar crystal planes to correct unfavorable thin film orientations (The XRD pattern is shown in Fig. 10f) [104].

Addressing defect control at the interface, the introduction of an ultrathin intermediate layer has demonstrated outstanding effects [107]. Guo et al. utilized atomic layer deposition (ALD) technology to coat a monoatomic layer of Al_2_O_3_, which not only passivated deep-level defects but also affected initial nucleation by altering substrate wettability [108]. This interface engineering is often combined with ion-assisted strategies; for instance, Geng et al., building upon this work, treated the Al_2_O_3_ interface layer with a NaOH solution, utilizing the synergistic effect of dual ions to suppress oxygen-induced unfavorable oriented growth and significantly optimize carrier dynamics (Fig. 10g) [105]. In substrate-configuration devices, interface engineering is primarily achieved through selenization or metal modification of the back electrode surface. In-situ surface selenization treatment of a tungsten (W) back contact can transition the growth mode from “layered” to “island-like”, successfully preparing nanorod arrays growing perpendicularly to the substrate [109]. In flexible devices (Fig. 10h), an in-situ grown MoSe_2_ layer utilizes its structural compatibility with Sb_2_S_3_ to induce the absorber layer to exhibit a [hk1] orientation and optimizes the band alignment at the back interface (Fig. 10i) [106]. In conclusion, interface engineering and chemical modification strategies have achieved a trinity synergistic integration of “orientation induction—defect passivation—band alignment”, providing a key pathway to break through the efficiency bottleneck of antimony-based solar cells.

### Post-treatment and thermal annealing strategies

During the preparation of Sb_2_(S,Se)_3_ thin films, the post-deposition treatment process is equally important, as it profoundly affects the crystallization quality, crystal orientation, and defect density of the thin films. Because the as-deposited precursor films typically exhibit an amorphous or weakly crystalline state, subsequent thermal annealing or chemical treatments not only provide the thermodynamic driving force for grain growth but also serve as the core window for breaking the transverse growth inertia of the quasi-one-dimensional [Sb_4_X_6_]_n_ (X = S, Se) segments and inducing their reconstruction into a vertical substrate orientation (the [hk1] direction).

#### Crystallization kinetics control via thermal field distribution

The post-annealing and selenization processes are not merely phase transition processes from precursors to a crystalline state, but more importantly, they are the decisive kinetic stages for regulating the alignment direction of the segments. Selenization temperature, as a fundamental parameter, determines the balance between crystallinity and orientation. Li et al. systematically investigated the effect of temperature on thin-film performance in a two-step method, discovering that as the temperature increases, although film compactness improves, the orientation exhibits significant temperature dependence: at higher temperatures, the intensity of the (hk0) plane is enhanced, while the (hk1) orientation conducive to transport is suppressed, and excessively high temperatures (> 360 °C) can lead to cracks in the film [110]. Studies by Costa et al. [111] and Kumar et al. [112] further confirmed that in post-treatments following thermal evaporation or sputtering, finding an appropriate temperature window is crucial. This is not only to ensure crystallization quality but also to kinetically compensate for selenium loss through co-evaporation or precise temperature control, thereby maintaining the growth momentum of the [hk1] orientation.

**Fig. 11:**
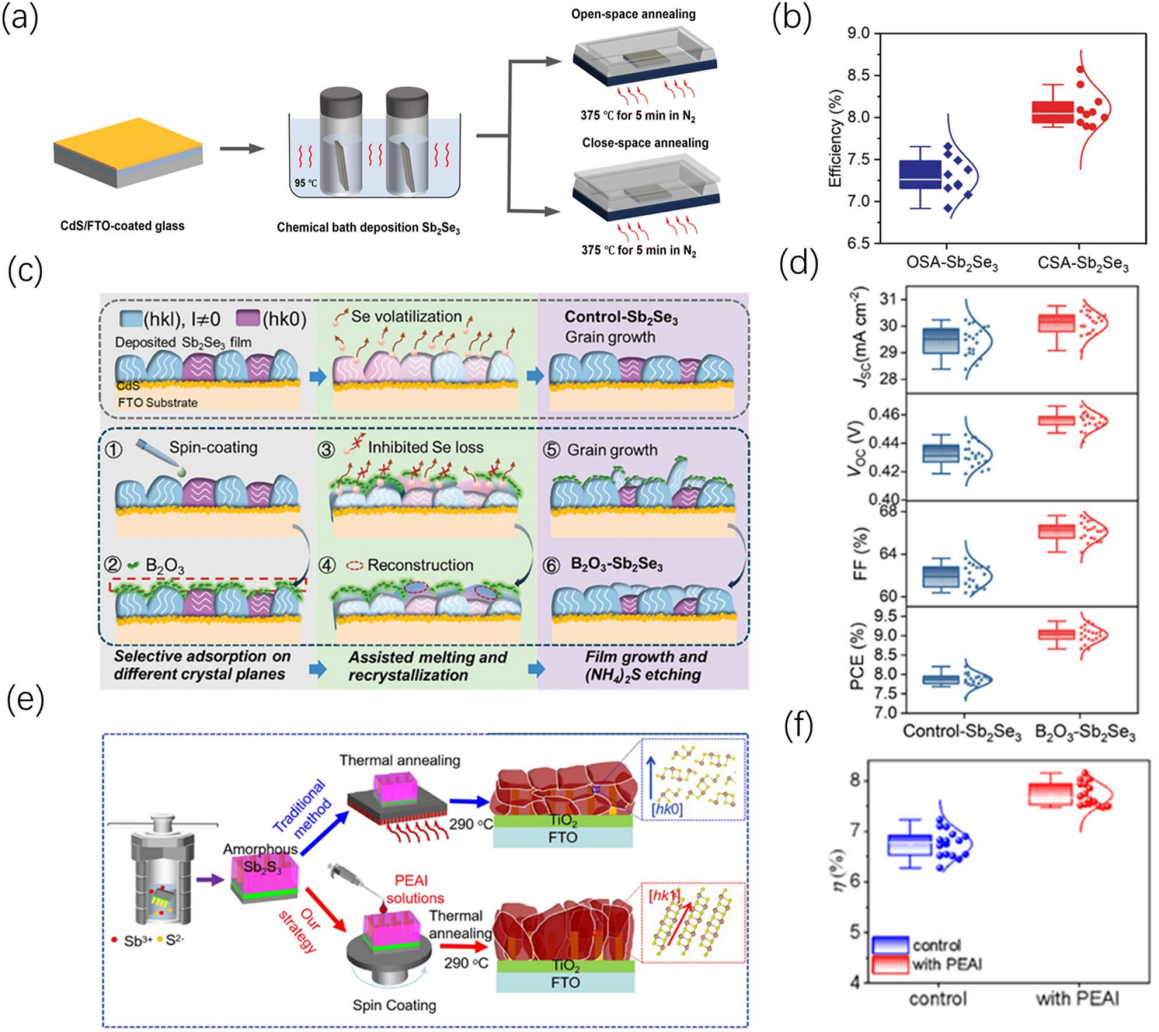
Comparison of post-treatment methods and their effects **a** Schematic illustration of the processes of treating Sb_2_Se_3_ thin films by close-space annealing (CSA) and open-space annealing (OSA). **b** Statistical box plots of efficiencies for the corresponding devices. Reproduced with permission [113]. Copyright 2025, Royal Society of Chemistry. **c** Schematic illustration of the crystallization processes of Control-Sb_2_Se_3_ and B_2_O_3_-Sb_2_Se_3_ thin films. **d** Comparison of performance parameters for the corresponding devices. Reproduced with permission [116]. Copyright 2024, Wiley–VCH GmbH. **e** Schematic illustration of the PEAI permeation modification treatment process, showing how molecules permeate along grain boundaries and induce columnar grain growth. **f** Statistical box plots of efficiencies for the corresponding devices. Reproduced with permission [117]. Copyright 2025, Wiley–VCH GmbH

Beyond the control of temperature intensity, the spatial distribution configuration of the thermal field directly affects the microenvironmental stability of the thin-film surface. Through a comparative study, Shen et al. found that traditional open-space annealing (OSA) struggles to suppress random nucleation due to large thermal gradients and unstable vapor pressure; in contrast, close-space annealing (CSA) provides a more uniform temperature gradient and stable selenium vapor pressure through a confined thermal field (Fig. 11a). This “confined kinetics” significantly altered the grain growth model, inducing a highly preferred [301] and [311] orientation in the film and boosting the device efficiency to 8.57% (Fig. 11b) [113]. Furthermore, Tang et al. adopted a similar concept in their post-selenization study following magnetron sputtering, utilizing a homogenized thermal field environment to synergistically enhance the lateral size and vertical alignment degree of grains, thereby significantly optimizing charge transport efficiency [114]. Additionally, selenization pressure is also a key parameter for regulating crystallization kinetics. Wen et al. found that precisely controlling the pressure to 15 mTorr during the post-selenization process can effectively balance the collision probability and kinetic energy of Se vapor, successfully inducing Sb_2_Se_3_ films with a highly preferred (001) vertical orientation. This kinetic regulation not only optimized the film orientation but also promoted the in-situ formation of a MoSe_2_ contact layer at the bottom, ultimately achieving an efficiency of 8.42% for the flexible solar cells [115].

#### Flux-assisted and preferred recrystallization strategies

The introduction of low-melting-point flux media can induce the secondary recrystallization of crystals, thereby forcibly breaking the thermodynamic intrinsic orientation limitations under non-equilibrium conditions. For example, Mao et al. proposed a highly inspiring molten-salt-assisted post-treatment interface strategy, providing a brand-new perspective for flux regulation. This research utilized the extremely high mobility and activity of molten-state ions to achieve the doping and surface modification of Sb_2_S_3_ thin films through a simple molten salt method. Under the molten salt environment, the rapid diffusion of high-energy ions not only induces a highly preferred (hk1) orientation but also achieves the densification and planarization of the thin-film surface. This recrystallization process not only optimizes the crystal growth pathway but also ingeniously shifts the valence band maximum (VBM) upward through lithium ion doping, thereby significantly suppressing charge recombination at the back-contact interface while reducing hole transport resistance [118]. Sheng et al. developed a B_2_O_3_-assisted post-annealing strategy. They discovered that low-melting-point B_2_O_3_ can preferentially adsorb onto the (hk1) crystal plane of Sb_2_Se_3_ and reduce its surface energy, thereby breaking the thermodynamic intrinsic limitations during the annealing process and inducing the thin film to undergo vertically oriented recrystallization (Fig. 11c). This strategy not only strengthens the vertical carrier transport channels but also substantially reduces selenium vacancy defects by suppressing Se volatilization, boosting the cell efficiency to 9.37% (Fig. 11d) [116]. The aforementioned studies demonstrate that the role of fluxes has evolved from single-aspect morphological induction into a multidimensional optimization mechanism integrating “orientation reshaping, energy level regulation, and interface passivation”. This recrystallization strategy based on non-equilibrium kinetics opens a new process pathway for developing low-cost, high-efficiency all-inorganic antimony-based solar cells.

#### Chemical permeation and ion regulation engineering

In recent years, synergistically optimizing the bulk phase and interface of thin films via the three-dimensional permeation effect of functional molecules or ions has evolved into a highly promising, omni-dimensional regulation approach [79]. During the film crystallization stage, introducing functional molecules with preferred adsorption characteristics can reshape crystal alignment habits from within the bulk phase; for example, Wang et al. employed a full-dimensional penetration strategy using degradable phenethylammonium iodide (PEAI). Through the directional binding between PEAI and the Sb_2_S_3_ (211) crystal plane, they induced the controlled growth of grains along the vertical direction (Fig. 11e) and simultaneously passivated deep-level defects, significantly improving the solar cell performance and efficiency (Fig. 11f) [117]. In addition to the penetration of organic macromolecules, the diffusion and penetration of inorganic halogen ions also demonstrate significant advantages in passivating deep-level intrinsic defects and reshaping crystal orientation. For example, Li et al. introduced a potassium iodide (KI) solution for the surface post-treatment of hydrothermally grown Sb_2_(S,Se)_3_ films. Driven by annealing, iodine ions diffuse and penetrate into the crystal lattice, occupying sulfur or selenium vacancies and fundamentally suppressing the formation of deep-level antimony antisite defects. This ion-level structural intervention not only optimized the energy band alignment at the interface but also significantly manipulated the crystal growth kinetics, successfully inducing the film to form a dense morphology with large grains and preferred growth along the [211] direction, which is favorable for carrier transport, ultimately boosting the power conversion efficiency of the device to 9.22% [119]. Building upon this, to address the energy level mismatch caused by internal compositional gradient mismatch, the aqueous selenium ion treatment (ASIT) technology developed by Chen et al. demonstrated the advantages of atomic-level compositional reconstruction. This technology utilized the ion permeation effect to achieve full-depth selenium enrichment of the thin film at room temperature, drastically reducing the interfacial valence band offset and establishing an ideal Type-II band alignment, thereby optimizing ultrafast charge transfer kinetics [120]. At the interface end, solution-based treatments similarly play an irreplaceable role; as shown by Adams et al., chemical washing and passivation using (NH_4_)_2_S or thioacetamide (TAA) solutions effectively removed residual oxide impurities on the Sb_2_Se_3_ surface and introduced a sulfur passivation layer to lower the interfacial barrier, significantly mitigating photovoltage loss [121]. In short, these chemical engineering strategies, ranging from bulk permeation to interface reconstruction, not only achieve the optimization of crystal orientation at lower temperatures but also synergistically resolve the inherent defect and energy level matching challenges of antimony-based chalcogenides through molecular- and atomic-scale structural interventions, providing a systematic solution for the fabrication of highly efficient and stable photovoltaic devices.

**Fig. 12:**
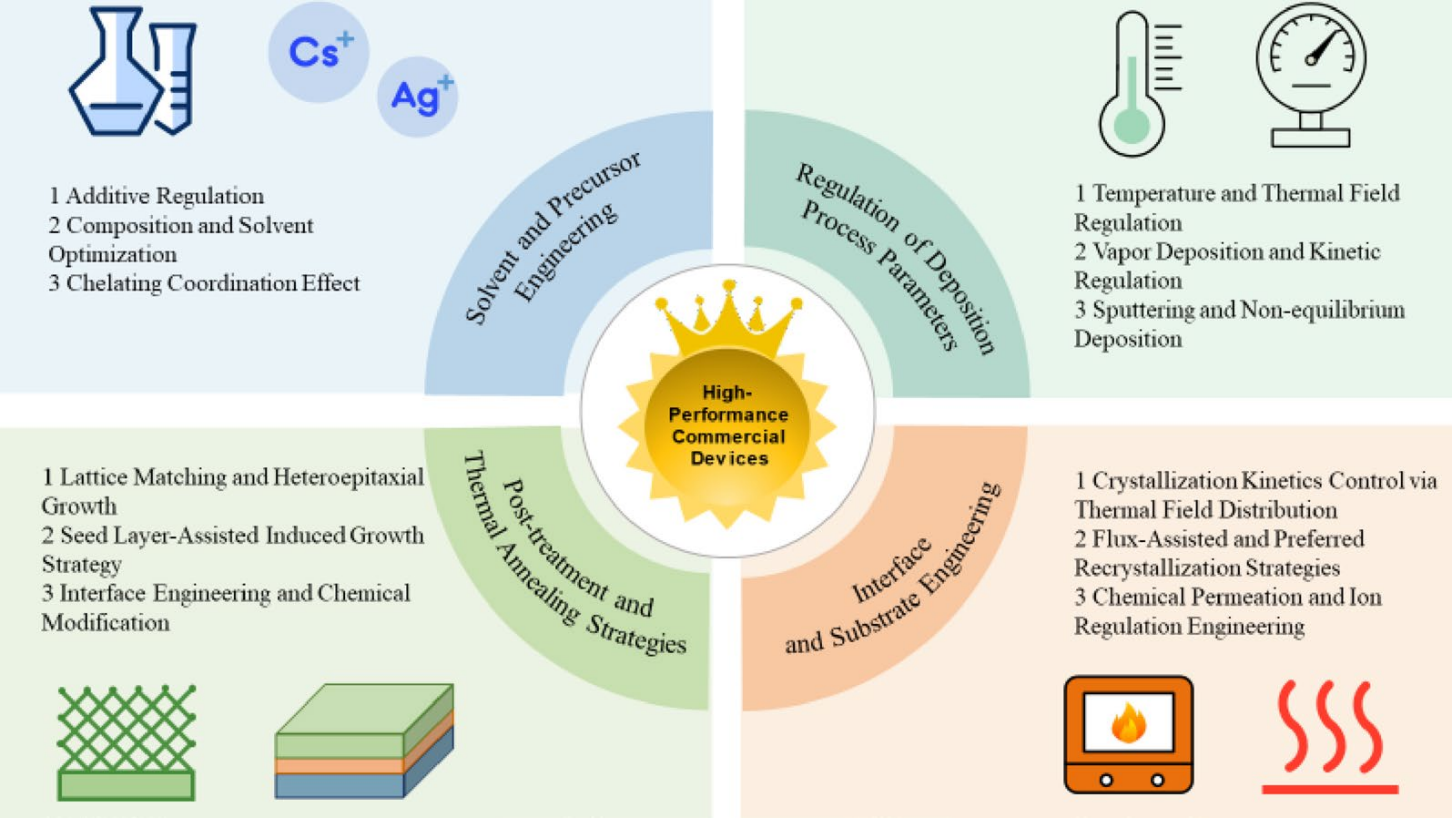
Summary of multi-dimensional orientation optimization strategies for high-performance antimony-based chalcogenide thin-film solar cells

**Fig. 13:**
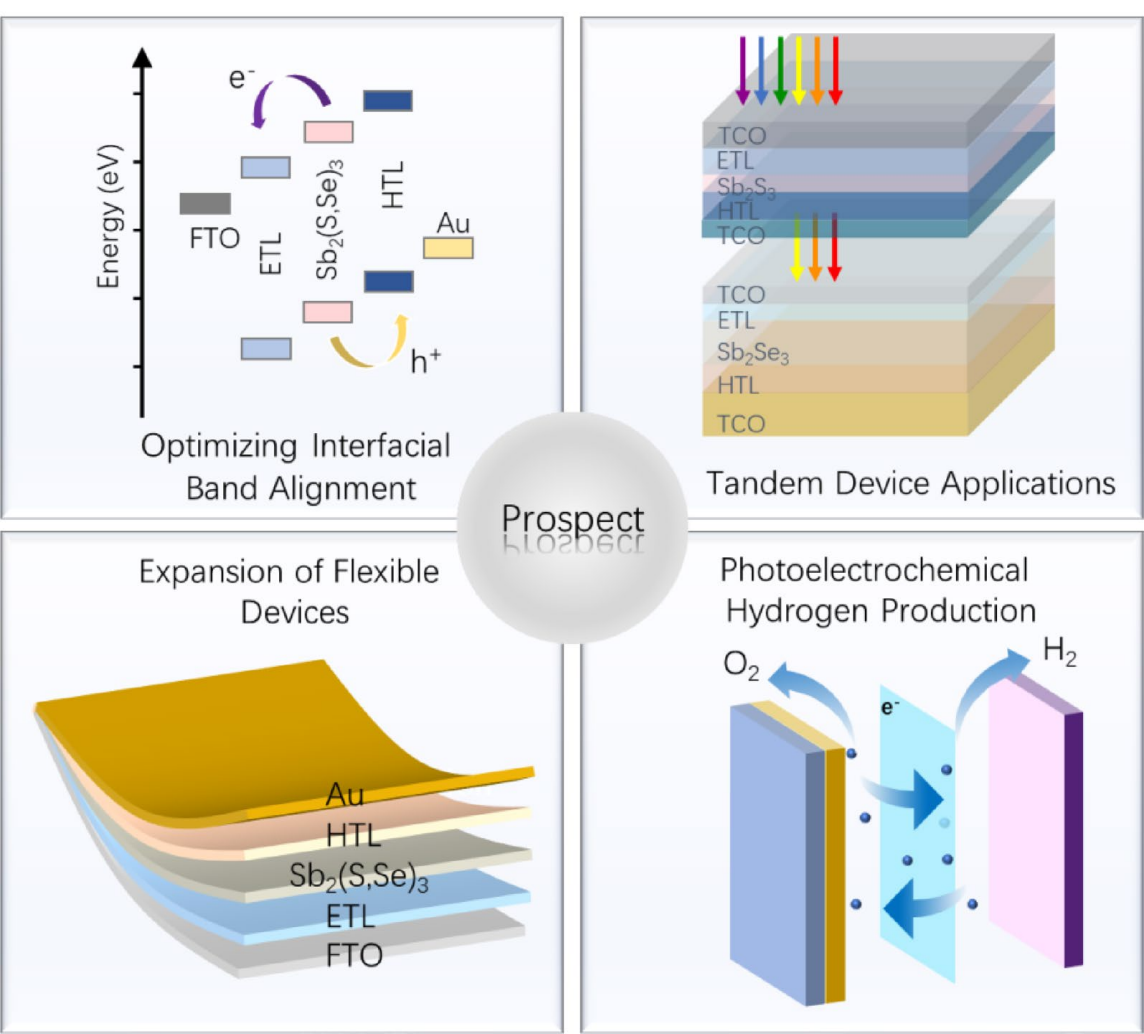
Future perspectives and emerging applications for antimony-based chalcogenide materials

To conclude, from the chemical regulation of precursor solutions and kinetic interventions in physical vapor deposition to heteroepitaxy at the substrate interface and post-deposition non-equilibrium reconstruction, researchers have conducted comprehensive explorations to break the intrinsic thermodynamic limitations of antimony-based materials and induce the [hk1] vertical orientation. These optimization strategies, which cover the entire nucleation and growth process, have not only successfully reshaped the micro-crystallographic features of the films but also synergistically addressed core physical challenges such as deep-level defects and energy band mismatches, thereby constructing highly efficient photoelectric transport pathways. To more intuitively illustrate the implementation of various orientation regulation strategies and their substantial contributions to device performance, Table 1 systematically summarizes the representative orientation optimization methods, the resulting preferred orientations, and the corresponding power conversion efficiencies of antimony-based chalcogenide thin-film solar cells reported in recent years. This summary aims to clearly delineate the current “structure-process-performance” relationship, providing a systematic technical framework and data reference for the future development of higher-efficiency antimony-based photovoltaic devices.

**Table 1:** Summary of orientation regulation strategies and performance of antimony-based chalcogenide thin-film solar cells

Strategy classification	Material system	Specific method	Preferred orientation	Device efficiency	References
Solvent and precursor engineering	Sb_2_(S,Se)_3_	Cs^+^ modulation	[211] [221]	8.46%	[70]
	Sb_2_S_3_	Synergistic Cl and Se doping	[211] [221]	7.15%	[71]
	Sb_2_(S,Se)_3_	In-situ passivation with sodium selenosulfate	[211] [221] [301]	10.81%	[31]
	Sb_2_(S,Se)_3_	Introduction of sodium borohydride (SB) additive	[211] [221] [301]	10.62%	[72]
	Sb_2_S_3_	Multi-sulfur source synergistic chemical bath deposition	[211] [221]	8.00%	[18]
	Sb_2_(S,Se)_3_	Sequential chemical bath deposition	[211] [221]	9.29%	[73]
	Sb_2_(S,Se)_3_	Solvent-assisted hydrothermal deposition (SHD)	[221]	10.75%	[75]
	Sb_2_S_3_	Solvent-assisted chemical bath deposition (S-CBD)	[211]	7.84%	[74]
	Sb_2_Se_3_	Thiourea (TU) complexation	[211] [221]	7.90%	[76]
	Sb_2_(S,Se)_3_	PO_4_^3−^ tetrahedral chelation	[211] [221]	10.67%	[78]
Deposition process parameter regulation	Sb_2_Se_3_	Rapid thermal evaporation to control substrate temperature	[211] [221] [301]	5.60%	[23]
	Sb_2_Se_3_	Close-space sublimation (CSS) combined with TiO_2_ interface engineering	[001]	9.20%	[11]
	Sb_2_(S,Se)_3_	Mean free path modulation (MFPM)	[211] [221] [301]	8.08%	[81]
	Sb_2_Se_3_	Molecular beam epitaxy (MBE)	[002] [101]	8.42%	[82]
	Sb_2_Se_3_	Vapor transport deposition combined with alloying composition control	[221]	7.60%	[24]
	Sb_2_S_3_	Vertical vapor transport deposition	[211] [221]	4.50%	[83]
	Sb_2_Se_3_	Injection vapor deposition (IVD)	[001]	10.12%	[25]
	Sb_2_(S,Se)_3_	Sequential co-evaporation deposition	[221]	8.00%	[84]
Interface and substrate engineering	Sb_2_(S,Se)_3_	Cd^2+^-induced epitaxial growth	[101] [211] [221]	9.20%	[88]
	Sb_2_(S,Se)_3_	MBE in-situ oxygen-doped CdS as electron transport layer	[211] [221] [301]	8.59%	[91]
	Sb_2_Se_3_	CBD Zn-doped C_x_Zn_1-x_S buffer layer	[211] [221] [301]	8.76%	[92]
	Sb_2_Se_3_	Spray pyrolysis preparation of ZnO buffer layer	[221]	5.93%	[93]
	Sb_2_S_3_	Heat-treated TiO_2_ inducing quasi-epitaxial growth of Sb_2_S_3_	[211] [221] [511]	5.40%	[95]
	Sb_2_Se_3_	Sb_2_S_3_ thin layer as seed layer	[002]	7.44%	[97]
	Sb_2_Se_3_	Introduction of seed layer on Mo substrate	[211] [221] [002]	8.50%	[98]
	Sb_2_S_3_	Oriented seed-assisted cyclic spin-coating annealing (RSCA)	[221]	5.15%	[99]
	Sb_2_S_3_	Micro-seed-assisted solution processing	[211]	5.70%	[100]
	Sb_2_(S,Se)_3_	High-crystallinity CSS CdS buffer layer	[221]	7.12%	[64]
	Sb_2_Se_3_	SbCl_3_ interface treatment of CdS	[002] [211] [221] [301] [321] [141]	6.89%	[102]
	Sb_2_Se_3_	Fluorine (F) doping regulation of CdS buffer layer	[211] [301]	9.30%	[103]
	Sb_2_(S,Se)_3_	BaBr_2_ regulation of heterogeneous nucleation	[211] [221]	10.12%	[104]
	Sb_2_(S,Se)_3_	Addition of buried single-atomic-layer Al_2_O_3_	[211] [221]	9.39%	[108]
	Sb_2_(S,Se)_3_	NaOH solution modified Al_2_O_3_ interface layer	[211] [221]	9.79%	[105]
	Sb_2_Se_3_	In-situ surface selenization treatment to form ultra-thin WSe_2_ layer	[101] [002]	8.46%	[109]
	Sb_2_S_3_	Back interface selenization treatment	[211]	3.75%	[106]
Post-treatment and Thermal annealing strategies	Sb_2_Se_3_	Co-evaporation and post-selenization treatment	[221] [321] [141] [041]	3.60%	[112]
	Sb_2_Se_3_	Close-space annealing (CSA)	[301] [311]	8.57%	[113]
	Sb_2_Se_3_	Magnetron sputtering and post-selenization treatment	[211] [221] [002]	6.06%	[114]
	Sb_2_Se_3_	Regulation of selenization kinetics	[001]	8.42%	[115]
	Sb_2_S_3_	Molten salt-assisted lithium doping	[211] [221]	6.16%	[118]
	Sb_2_Se_3_	B_2_O_3_-assisted post-annealing	[211] [321]	9.37%	[116]
	Sb_2_S_3_	PEAI pre-treatment assisted full-dimensional penetration strategy	[111] [211] [221] [301] [501]	8.21%	[117]
	Sb_2_(S,Se)_3_	Potassium iodide (KI) surface post-treatment	[211] [221] [301]	9.22%	[119]
	Sb_2_(S,Se)_3_	Aqueous selenium ion engineering (ASIT)	[221]	10.38%	[120]

## Conclusion and prospect

### Conclusion

Antimony-based chalcogenides (Sb_2_S_3_, Sb_2_Se_3_, and their alloy Sb_2_(S,Se)_3_) represent a representative class of quasi-one-dimensional semiconductor materials where unique photoelectric anisotropy acts as both a primary performance bottleneck and a key pathway to high efficiency. At the fundamental physical and growth mechanism levels, the distinct “strong intra-chain vs. weak inter-chain” bonding dictates this transport anisotropy, the [hk1] orientation along covalent bonds offers high carrier mobility and “self-passivating” benign grain boundaries, whereas the thermodynamically preferred [hk0] orientation across van der Waals gaps induces severe recombination and potential barrier hopping. Consequently, the core scientific challenge lies in breaking the thermodynamic equilibrium of surface energy minimization through kinetic interventions, thereby inducing the nucleation and sustained growth of the metastable [hk1] vertical orientation.

To achieve this crucial microstructural transition, this review has systematically summarized four effective regulation pathways (Fig. 12). Specifically, solvent and precursor engineering precisely regulate liquid-phase nucleation kinetics via ion mediation, compositional adjustment, and chelating coordination. In parallel, deposition process optimization enables the shift from thermodynamic to kinetic control in vapor deposition by tuning temperature, background pressure, and deposition rates. Furthermore, interface and substrate engineering achieve vertical anchoring of the [hk1] orientation by lowering interfacial energy through lattice-matched epitaxy, seed layer induction, and chemical modifications. Finally, post-treatment reconstruction repairs orientation deviations and passivates defects via thermal field regulation, flux-assisted recrystallization, and ion penetration engineering. It is precisely thanks to the synergistic application of these multi-dimensional strategies that the power conversion efficiency of antimony-based thin-film solar cells has successfully breached the 10% mark, unequivocally verifying the decisive role of crystal orientation regulation in approaching the theoretical performance limits of these promising photovoltaic devices.

### Prospect

Although significant progress has been made in crystal orientation regulation, the efficiency of antimony-based chalcogenide photovoltaic devices still lags far behind their Shockley-Queisser theoretical limit (> 30%) [122]. To further unleash their potential, future research needs to continue deepening in the following dimensions (Fig. 13):

1. Optimizing Interfacial Band Alignment
  Current orientation modulation primarily focuses on the absorber layer itself; however, the interfacial band alignment between the electron transport layer (ETL) and the absorber layer is equally crucial. Future research should be dedicated to constructing high-quality heterojunction interfaces with “orientation inheritance” to mitigate interfacial lattice mismatch and non-radiative recombination, which serves as a vital direction for enhancing the fill factor of the devices. Furthermore, exploring novel wide-bandgap buffer materials to replace traditional CdS, thereby improving spectral response and optimizing interfacial band alignment, represents an avenue worthy of in-depth exploration [93, 123].
2. Tandem Device Applications
  Beyond single-junction solar cells, antimony-based materials demonstrate significant advantages in tandem cell applications, owing to their suitable bandgaps and the high carrier mobility achieved through orientation modulation. Utilizing them as bottom cells in tandem solar cells[124], paired with wide-bandgap top cells, will be a highly promising development trajectory to surpass the theoretical efficiency limits of single-junction devices and realize more efficient full-spectrum absorption.
3. Expansion of Flexible Devices
  Benefiting from the remarkable mechanical stability of antimony-based materials and the benign grain boundary characteristics induced by the [hk1] orientation, these materials possess intrinsic advantages in the flexible photovoltaics sector [115, 125]. Future studies must further investigate low-temperature processes to achieve high-quality vertically oriented growth on flexible substrates (such as metal foils and polymers). Concurrently, resolving the uniformity of thermal and flow fields during large-area fabrication will be pivotal in propelling this material from laboratory-scale cells toward commercial flexible module applications.
4. Photoelectrochemical Hydrogen Production
  Capitalizing on their suitable bandgaps and optimized carrier transport channels, antimony-based thin films also exhibit tremendous potential in the field of photoelectrochemical (PEC) water splitting for hydrogen production. Tailoring specific surface orientations to enhance catalytic reaction kinetics poses a novel challenge for future PEC applications. Meanwhile, employing protective overlayer technologies to address the long-term photocorrosion degradation of these materials in aqueous environments will remain a core focus for expanding their utilization in photoelectrochemical catalysis.

In summary, by deeply understanding crystal growth kinetics and combining multi-dimensional fine regulation strategies, we have reason to believe that antimony-based chalcogenides will occupy an important place in the future fields of thin-film photovoltaics and photodetection.

## Data Availability

The datasets used and/or analysed during the current study are available from the corresponding author on reasonable request.
